# Acetyltransferase in cardiovascular disease and aging

**DOI:** 10.20517/jca.2024.21

**Published:** 2024-12-31

**Authors:** Mariko Aoyagi Keller, Michinari Nakamura

**Affiliations:** Department of Cell Biology and Molecular Medicine, Rutgers New Jersey Medical School, Newark, NJ 07103, USA.

**Keywords:** Acetyltransferase, acetylation, lysine acetyltransferase, KAT, acetyl coenzyme A, deacetylase, cardiovascular disease, aging, diet

## Abstract

Acetyltransferases are enzymes that catalyze the transfer of an acetyl group to a substrate, a modification referred to as acetylation. Loss-of-function variants in genes encoding acetyltransferases can lead to congenital disorders, often characterized by intellectual disability and heart and muscle defects. Their activity is influenced by dietary nutrients that alter acetyl coenzyme A levels, a key cofactor. Cardiovascular diseases, including ischemic, hypertensive, and diabetic heart diseases - leading causes of mortality in the elderly - are largely attributed to prolonged lifespan and the growing prevalence of metabolic syndrome. Acetyltransferases thus serve as a crucial link between lifestyle modifications, cardiometabolic disease, and aging through both epigenomic and non-epigenomic mechanisms. In this review, we discuss the roles and relevance of acetyltransferases. While the sirtuin family of deacetylases has been extensively studied in longevity, particularly through fasting-mediated NAD^+^ metabolism, recent research has brought attention to the essential roles of acetyltransferases in health and aging-related pathways, including cell proliferation, DNA damage response, mitochondrial function, inflammation, and senescence. We begin with an overview of acetyltransferases, classifying them by domain structure, including canonical and non-canonical lysine acetyltransferases, N-terminal acetyltransferases, and sialic acid *O*-acetyltransferases. We then discuss recent advances in understanding acetyltransferase-related pathologies, particularly focusing on cardiovascular disease and aging, and explore their potential therapeutic applications for promoting health in older individuals.

## Graphical Abstract

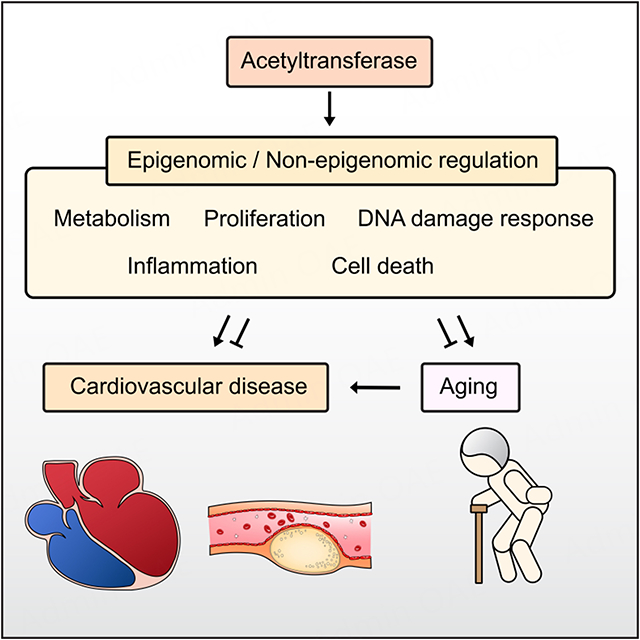

## INTRODUCTION

According to data from the World Health Organization, global life expectancy at birth increased from 66.8 years in 2000 to 73.1 years in 2019, marking a 6.3-year rise before the COVID-19 pandemic. Healthy life expectancy also improved by 9%, increasing from 58.1 years in 2000 to 63.5 years in 2019, primarily due to declining mortality rates. However, the high morbidity and mortality of cardiovascular disease remain critical issues, driven in part by aging and the growing prevalence of metabolic disorders linked to socioenvironmental changes^[1,2]^. Unhealthy dietary patterns, such as the consumption of high-fat and ultra-processed foods, along with sedentary lifestyles, dramatically impact circulating metabolite profiles and disrupt cellular metabolism, contributing to the development of cardiometabolic disease^[3,4]^. Understanding how biological functions are altered in the elderly or individuals with metabolic syndrome and how these changes contribute to cardiovascular disease could provide valuable insights for developing novel therapeutics to maintain cardiovascular health.

**Figure 1.**
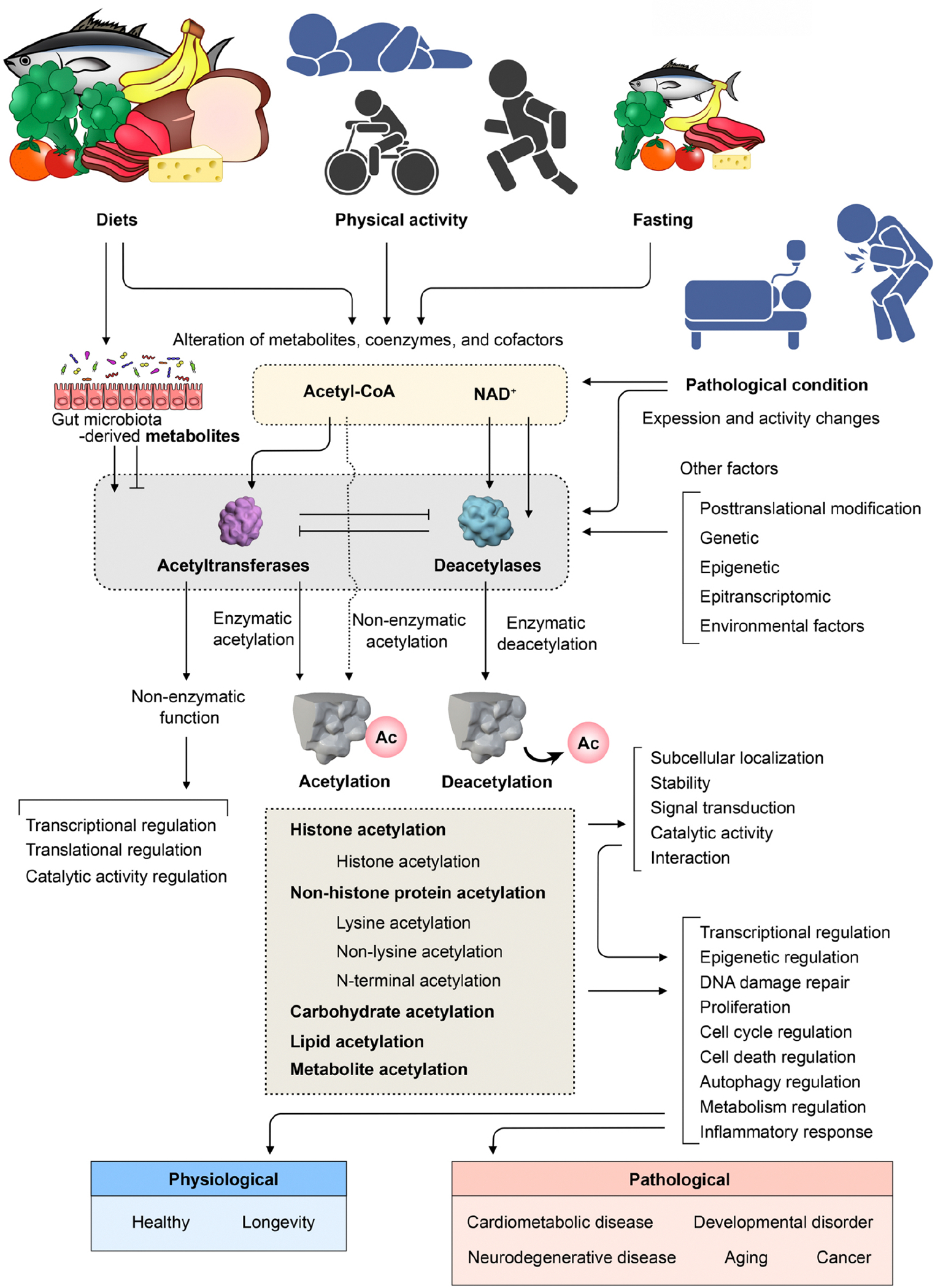
Acetyltransferases and deacetylases in the regulation of acetylation and deacetylation through acetyl-CoA and NAD^+^. Diets, physical activity, and fasting influence the levels of acetyl-coenzyme A (acetyl-CoA) and NAD^+^, which modulate the activities of acetyltransferases and deacetylases, mediating acetylation and deacetylation, respectively. This is also interdependently regulated by one another, highlighting the intricate crosstalk between acetyltransferases and deacetylases in maintaining cellular homeostasis. Pathological conditions (e.g., cardiometabolic disease, cancer, and aging) and other factors (e.g., post-translational modifications, genetic, epigenetic, epitranscriptomic, and environmental factors) also impact the enzymatic activities of these proteins. Additionally, acetyl-CoA can drive non-enzymatic acetylation. Acetylation regulates diverse cellular processes, including transcription, epigenetics, DNA damage repair, proliferation, cell death, cell cycle progression, autophagy, cellular metabolism, and inflammatory responses. This regulation occurs through the control of signal transduction, subcellular localization, protein stability, catalytic activity, and protein-protein interactions. Collectively, these processes underscore the essential role of acetyltransferase-mediated acetylation and deacetylase-mediated deacetylation in maintaining healthy longevity or contributing to pathological conditions, such as cardiometabolic diseases, neurodegenerative disorders, and aging.

Nutritional status and physical activity influence the balance of intracellular levels of key cofactors, acetyl-Coenzyme A (acetyl-CoA) and nicotinamide adenine dinucleotide (NAD^+^), which regulate the activity of acetyltransferases and deacetylases - enzymes that add or remove acetyl groups on histone and non-histone targets^[5,6]^ [Figure 1]. These cellular processes are known as acetylation and deacetylation, respectively^[7,8]^. Acetylation is a highly regulated, reversible, and dynamic post-translational modification (PTM) that not only controls chromatin structure and function, often associated with transcriptional activation, but also regulates signaling pathways involved in cellular metabolism, proliferation, DNA damage response, and cell death. Abnormal acetylation of histone and non-histone proteins due to dysregulation of acetyltransferases and deacetylases is implicated in various human diseases, including cancer, cardiometabolic diseases, congenital disorders, neurodevelopmental disorders, and aging-related organ dysfunction.

Aging is one of the major risk factors for cardiovascular diseases. Impaired DNA damage repair mechanisms lead to epigenetic alterations and mitochondrial dysfunction, which drive inflammation and dysregulated signaling pathways, contributing to aging-related chronic conditions such as cardiometabolic disease and cancer^[9]^. Dietary restriction is known to extend lifespan across various animal species, with intermittent fasting or moderate low-carbohydrate high-fat diets gaining much attention for their therapeutic benefits in cardiovascular and aging-related diseases. While histone or lysine deacetylases (HDACs or KDACs), including sirtuins, which depend on NAD^+^ or Zn^2+^, have been extensively studied for their roles in longevity, recent studies have uncovered molecular mechanisms by which acetyltransferases regulate protein function and cellular metabolism, highlighting their significance in these pathologies. In this review, we begin by introducing the classification of acetyltransferases and then explore recent advances in understanding their pathological roles in cardiovascular disease and aging. We also discuss their therapeutic potential and applications as biomarkers.

## CLASSIFICATION OF ACETYLTRANSFERASES

Acetylation is catalyzed by acetyltransferases, which transfer acetyl groups from acetyl-CoA to specific substrates. Lysine acetylation can also occur non-enzymatically through a chemical reaction involving the metabolites acetyl-phosphate or acetyl-CoA. Acetyltransferases are classified based on their domain structural homology and substrate specificity. In this review, we classify acetyltransferases into four groups: (canonical) lysine acetyltransferases (KATs), non-canonical KATs, N-terminal acetyltransferases (NATs), and others, such as sialic acid *O*-acetyltransferases (SOATs). The numbering of KATs is based on the order in which their acetyltransferase activity was discovered, irrespective of species, and follows the sequence of published reports^[10]^. When an alternative name is more commonly used in the literature due to its biological or functional role in a protein or its complex, we will reference that term initially. It is important to note that other acetyltransferases, such as acetyl-CoA C-acetyltransferase (ACAT) and glycine C-acetyltransferase (GCAT), which both utilize acetyl-CoA as a donor of acetyl groups, are not included in this classification because their primary function lies in metabolic processes related to energy production, targeting acetyl-CoA or small molecules (e.g., glycine or lipids) as substrates, rather than modifying proteins or histones.

### a: Canonical lysine acetyltransferases

**Figure 2.**
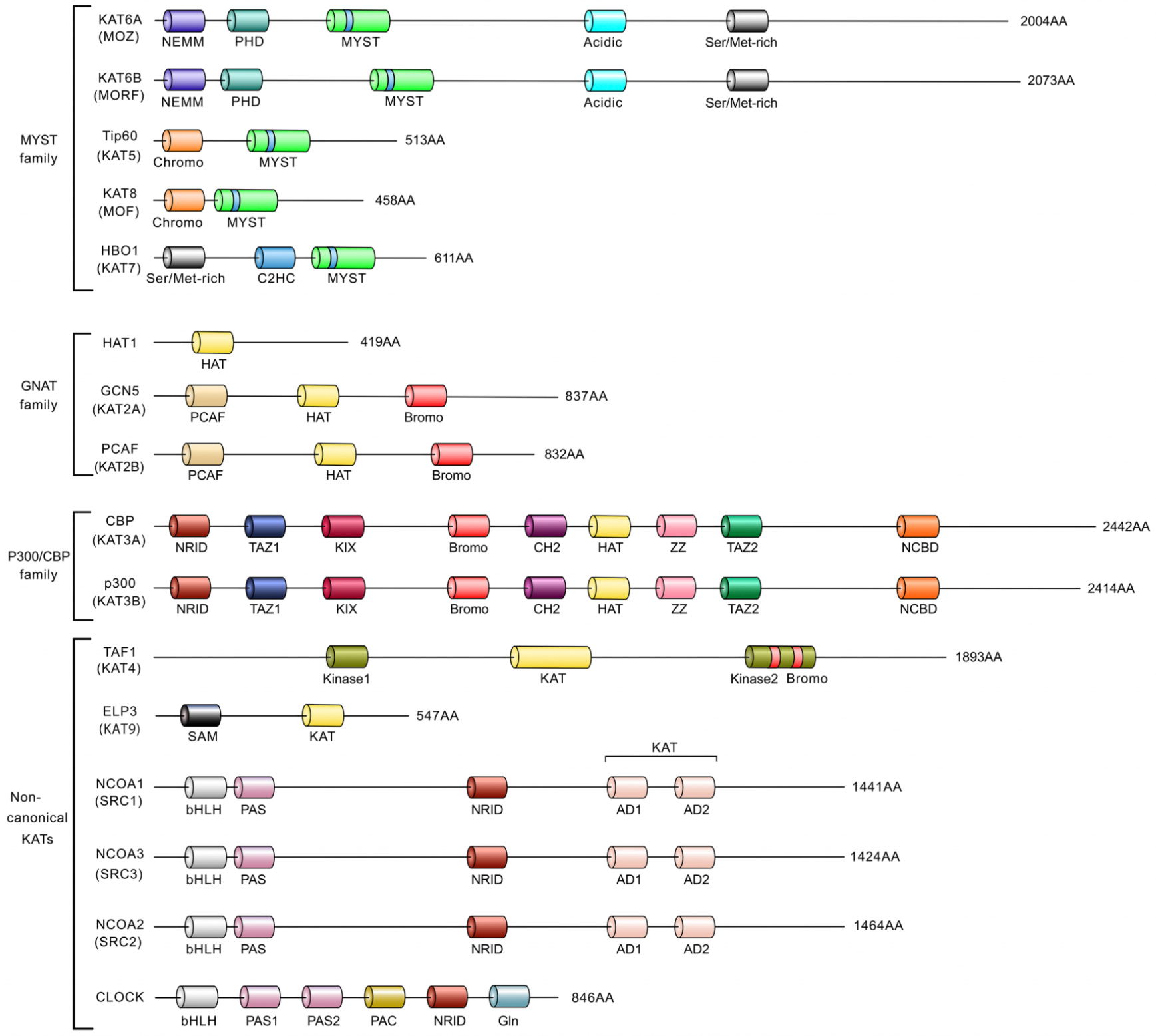
Domain structure of canonical and non-canonical acetyltransferases. Acetyltransferases are classified based on their domain structural homology and substrate specificity. Canonical lysine acetyltransferases (KATs) are further grouped into three major families: the MYST family consisting of five members [KAT6A (MOZ), KAT6B (MORF), Tip60 (KAT5), KAT8 (MOF), and HBO1 (KAT7)], the GNAT family consisting of three members [HAT1, GCN5 (KAT2A), and PCAF (KAT2B)], and the p300/CBP family consisting of two members [CBP (KAT3A) and p300 (KAT3B)]. Non-canonical KATs are categorized into two primary groups: transcription coactivator-related KATs consisting of three members [TAF1 (KAT4), ELP3 (KAT9), and GTF3C4 (KAT12)] and steroid receptor coactivator-related KATs consisting of four members (NCOA1 (SRC1), NCOA3 (SRC3), NCOA2 (SRC2), and CLOCK). The number of amino acids (AA) for each human protein is listed. NEMM, N-terminal part of Enok, MOZ or MORF; PHD, PHD zinc figure; MYST, MYST-type KAT domain including zinc figure domain (blue); Acidic, acidic domain; Ser/Met-rich, serine/methionine-rich domain; Chromo, chromodomain; C2HC, C2HC-type zinc figure domain; HAT, GNAT or p300/CBP-type histone acetyltransferase domain; NRID, N-terminal nuclear receptor interaction domain; TAZ, transcription adaptor putative zinc figure domain; KIX, kinase-inducible domain (KID) interacting domain; CH2, cysteine/histidine-rich zinc figure domain; ZZ, ZZ-type zinc finger domain; NCBD, nuclear coactivator binding domain; Kinase, kinase domain; Bromo, bromodomain; SAM, S-adenosylmethionine (SAM) domain; bHLH, basic helix-loop-helix domain; PAS, Per/Arnt/Sim domain; AD, transcriptional activation domain; PAC, C-terminal to a subset of PAS motifs; Gln, glutamine-rich C-terminal domain.

Canonical lysine acetyltransferases (KATs) transfer an acetyl group from acetyl-CoA to the primary amine in the ε-position of the lysine side chain within proteins, including both histones and non-histones. The transfer of an acetyl group onto specific lysine residues results in a loss of positive charge on histones, thereby destabilizing the histone-DNA interaction, increasing chromatin accessibility for RNA polymerases and transcription factors, and enhancing translation. Besides regulating gene expression, histone acetylation is also essential for DNA replication and repair^[11]^. KATs were initially classified into two groups, type A and B KATs, based on subcellular localization and substrate specificity^[12]^. Type A KATs are mainly found in the nucleus and acetylate histones and other chromatin-associated proteins in concert with specific DNA-binding transcriptional activators, thereby directly influencing transcription. In contrast, Type B KATs are predominantly localized in the cytoplasm and facilitate the acetylation process of newly synthesized histones, thereby impacting their assembly and structure of the nucleosome. However, subsequent studies, as discussed later, have reported the subcellular localization and functions of several KATs that deviate from the initial Type A and Type B classification, making it challenging to categorize all KATs within this framework. Therefore, the Type A and Type B classification is not applied here. Canonical KATs are grouped into three major families based on their catalytic and other domain structure and substrate specificity^[10,13]^ [Figure 2]: the MYST family, the Gcn5-related N-acetyltransferase (GNAT) family, and the p300/CREB-binding protein (CBP) family.

### MYST family

The MYST family comprises five members: KAT6A, KAT6B, KAT5, KAT7, and KAT8. While these KATs share the conserved MYST catalytic domains necessary for acetyltransferase activity, they exhibit substrate specificity toward histones with distinct epigenetic marks and various non-histone proteins.

### KAT6A

KAT6A, also known as MOZ (monocytic leukemia zinc finger protein) or MYST3, is a member of the MYST family of histone acetyltransferases^[14]^. Structurally, it contains a MYST-type KAT domain responsible for its acetyltransferase activity, along with zinc finger motifs, an acidic (glutamate/aspartate-rich) domain, and a serine/methionine-rich domain in the C-terminal region^[14,15]^. KAT6A is predominantly localized in the nucleus, where it plays a vital role in gene expression, cell cycle progression, and hematopoiesis [Table 1]. It is also found in the cytosol. KAT6A mainly acetylates histone H3 at lysine 9 (H3K9) and lysine 14 (H3K14)^[16]^. In addition, KAT6A acetylates the tumor suppressor protein p53 at K120 and K382, promoting complex formation with p53 in response to irradiation-induced DNA damage in MCF-7 cells^[17]^. This interaction enhances p53 transcriptional activity, leading to the selective induction of p21 expression, which in turn causes cell cycle arrest^[18]^. KAT6A-deficient mouse embryonic fibroblasts (MEFs) showed impaired p53-mediated expression of the *Cyclin-Dependent Kinase Inhibitor 1A* (*CDKN1A*, encoding p21) gene and failed to arrest in the G1 phase, suggesting that the loss of KAT6A-p53 complex-mediated p21 expression contributes to tumor pathogenesis and leukemogenesis.

KAT6A knockout (KO) mice, which lack exon 2 containing the first ATG start codon, die around embryonic day (E)15, exhibiting a severe deficit in hematopoietic stem cells (HSCs)^[19]^. Similarly, mice with a mutation that specifically lacks acetyltransferase activity, by replacing glycine (G) 657 with glutamic acid in the KAT6A catalytic domain, exhibit marked reductions in HSC numbers^[20]^. These results indicate that KAT6A and its acetyltransferase activity are required for maintaining HSC functionality. Homozygous KAT6A C-terminal truncated mutant mice, created by in-frame insertion of a neomycin cassette into exon 16, die at birth, likely due to aortic arch defects, with facial abnormalities^[21]^. On embryonic day 18.5, these homozygous mutant mice exhibit severe dysgenesis of both the thymus and spleen, along with profound defects in HSCs functionality. KAT6A C-terminal truncated mutant mice also display hypoacetylation of H3K9, which is relatively specific at *Hox* gene loci, causing repression of *Hox* gene expression and anterior homeotic transformation, characterized by the presence of an additional 8th cervical vertebra. These phenotypes can be reversed by retinoic acid, a potent activator of *Hox* gene expression^[22]^. KAT6A lymphocyte-specific knockout (KO) mice, generated using lymphocyte-specific protein tyrosine kinase (LcK)-Cre, exhibit reduced H3K9 acetylation (H3K9ac) at the *Cd8* gene locus and lower *Cd8α* transcript levels^[23]^. Furthermore, a recent study showed that KAT6A deficiency impairs synaptic structure and plasticity in the hippocampal CA3 region, but not in CA1, resulting in memory deficits in mice. This was shown using NEX-Cre mice, which express Cre recombinase in pyramidal neurons of the neocortex and hippocampus^[24]^. This study also generated 3x hemagglutinin (HA)-tagged KAT6A knockin mice and revealed that KAT6A directly targets the promoter region of *R-spondin 2* (*Rspo2*), a Wnt/β-catenin signaling activator, regulating its transcription by modulating H3K23 acetylation (H3K23ac) in hippocampus, as shown by RNA-sequencing and chromatin immunoprecipitation-qPCR assay.

### KAT6B

KAT6B, also referred to as MORF (MOZ-related factor) or MYST4, is a member of the MYST family of KATs^[25]^. Structurally, KAT6B contains a MYST-type KAT domain responsible for its acetyltransferase activity, zinc finger motifs, an acidic (glutamate/aspartate-rich) domain, and a serine/methionine-rich domain. Predominantly localized in the nucleus, KAT6B plays essential roles in gene expression, development, and differentiation [Table 1]. It primarily acetylates H3K14 and H3K23^[26]^. KAT6B forms a complex with KAT6A, ING5 (inhibitor of growth 5), and bromodomain-PHD finger-containing protein1 (BRPF1), which is linked to multiple cancers and genetic disorders associated with intellectual disability.

Genetic haploinsufficiency of KAT6B was identified in a patient with a Noonan syndrome-like phenotype with reduced H3 acetylation, though this is not the typical genetic mutation associated with the syndrome^[27]^. Homozygous deletion of Querkopf, the mouse homolog of human KAT6B, results in craniofacial abnormalities and defects in central nervous system development in mice^[28]^, resembling human phenotypes such as growth retardation, facial dysmorphism, and brain anomalies seen in Noonan syndrome^[27]^. Additionally, whole-exome sequencing has identified mutations in the *KAT6B* gene in patients with the Say-Barber-Biesecker variant of Ohdo syndrome (SBBYSS), characterized by severe intellectual disability, blepharophimosis, a mask-like facial appearance, and autism-like behaviors^[29]^. Mutations in the *KAT6B* gene have also been found in patients with Genitopatellar syndrome (GPS), characterized by skeletal dysplasia and cerebral and genital anomalies^[30]^. While most GPS variants are observed in the last coding exon of KAT6B, about half of SBBYSS variants are located in more proximal exons, indicating that the location of these mutations in the *KAT6B* gene may contribute to the phenotypic differences between SBBYSS and GPS.

KAT6B homozygous KO mice die before weaning^[31]^. On embryonic day 18.5, homozygous and heterozygous KO mice exhibit 49% and 18% reductions in H3K9ac in the cortex relative to controls, respectively. Although H3K23ac decreases by 12% in the homozygous KO cortex, H3K14ac remained unchanged, indicating that H3K9 is a major target of KAT6B. Heterozygous KAT6B KO mice exhibit lower body weight, reduced vocalization, delayed auditory startle response, and learning and memory deficits, which can be partially restored by postnatal therapeutic intervention with valproic acid, a histone deacetylase inhibitor, or acetyl-carnitine (ALCAR), an acetyl donor^[31]^. A recent study using HSCs with genetic inhibition of KAT6B and/or KAT6A further demonstrated that KAT6B and KAT6A cooperatively stimulate transcription of genes involved in hematopoiesis, including the Hoxa cluster, *Pbx1*, *Meis1*, Gata family, *Erg*, and *Flt3*, thereby supporting HSC development and function^[32]^.

### Tip60

Tip60 (Tat interactive protein, 60 kDa), also known as KAT5, is a member of the MYST family of KATs. Structurally, Tip60 features a MYST-type KAT domain responsible for its acetyltransferase activity, a chromodomain for recognizing methylated histones, and zinc finger motifs for DNA binding. Tip60 is primarily localized in the nucleus, where it functions as part of the multisubunit NuA4 complex [Table 1]. Initially identified as a cofactor specifically interacting with the human immunodeficiency virus (HIV-1) Tat protein, an activator of HIV gene expression^[33]^, Tip60 was later recognized as a substrate-specific histone acetyltransferase with an evolutionarily conserved domain shared with yeast silencing factors^[34]^. Tip60 acetylates several histone residues, including H3K14, H4K5, H4K8, H4K12, H4K16, and H2A at K5, as determined by matrix-assisted laser desorption/ionization mass spectrometry (MALDI-MS) measurements and Lys-C endopeptidase digestion, or by incorporating radiolabeled acetate into synthetic peptides^[35]^. In addition to histones, Tip60 has various non-histone targets. For example, Tip60 interacts with and acetylates p53 at K120, located in the DNA-binding domain, enhancing p53-dependent activation of *p21* and *puma*, which promotes p53-mediated apoptotic responses, DNA damage response, and tumor suppression^[36]^. Tip60 also acetylates c-Myc, as measured by nanoelectrospray tandem mass spectrometry, dramatically increasing c-Myc protein stability^[37]^.

Homozygous deletion of the *Tip60* gene, achieved by replacing exons 1–9 with a neomycin resistance cassette, results in embryonic lethality near the blastocyst stage of development^[38]^. GTEx datasets show that Tip60 is ubiquitously expressed at various levels across multiple organ systems^[39]^. Tip60 haploinsufficiency accelerates Myc-induced lymphomagenesis in mice by abrogating Myc-induced DNA damage response signaling^[40]^. Consistent with this, ectopic expression of a mutant Tip60 lacking KAT activity impairs DNA repair and the apoptotic response to γ-irradiation-induced DNA damage in cells^[41]^. Comparative analysis reveals significantly lower expression of Tip60 in lung adenocarcinoma relative to normal lung tissues, highlighting its importance in lung cancer tumorigenesis^[39]^. However, further inhibition of Tip60 specifically in alveolar epithelial cells suppressed tumorigenesis in a lung-specific EGFR-L858R-T790M transgenic lung cancer mouse model without affecting normal lung homeostasis, indicating that Tip60 expression is crucial for lung tumorigenesis. These findings underscore the cell-autonomous and context-specific roles of Tip60.

Tip60 is highly expressed in the hippocampal CA1 region, which is critical for memory function, alongside KAT2A^[42]^. Hippocampus-specific deletion of Tip60 using the tamoxifen-inducible *CaMKCreER*^*T2*^ driver line exhibits extensive neurodegeneration and reduced H4K12 acetylation levels in the CA region, leading to memory impairment and behavioral abnormalities^[43]^. Neural stem/progenitor cell (NSC)-specific deletion of Tip60 results in perinatal lethality^[44]^. The deletion of Tip60 in NSCs causes severe brain hypoplasia and microcephaly with abnormalities in neurogenesis and accumulation of M phase cells, with reduced levels of H4K8ac and H4K12ac. Recently, three individuals with heterozygous *de novo* missense mutations in Tip60 have been reported, displaying cerebral malformations, progressive cerebellar atrophy, seizures, global developmental delay or intellectual disability, and severe sleep disturbances, along with reduced acetylation activity at the histone H4 tail in chromatin^[45]^. These findings collectively underscore the essential role of Tip60 in DNA damage response, neurogenesis, and neuronal functions.

### KAT8

KAT8, also known as MOF (males absent on the first) or MYST1, is a key member of the MYST family of KATs. Initially, KAT8 was recognized for its role in the X chromosome dosage compensation system^[46]^. Structurally, KAT8 contains an amino-terminal chromobarrel and a central MYST-type KAT domain. It specifically acetylates H4K16^[47]^. Predominantly localized in the nucleus, KAT8 plays a crucial role in chromatin remodeling and gene expression [Table 1]. In addition to histones, KAT8 acetylates several non-histone substrates, including p53. KAT8 and Tip60 acetylate p53 at K120 in response to genotoxic stresses in H1299 cells, promoting p53-mediated apoptosis by stimulating *Bax* and *Puma* gene expression^[48]^. Expression of an acetylation-resistant (K120R) p53 mutant or lentivirus-mediated suppression of KAT8 and Tip60 reduces the ability of p53 to induce Bax and Puma in cells *in vitro*^[48]^.

KAT8 also regulates oxidative stress and drug resistance by interacting with and acetylating Nrf2 at K588, facilitating its nuclear retention and activation of Nrf2-dependent gene expression in MEFs and A549 human lung cancer cells^[49]^. Additionally, KAT8 acetylates TIP5, a subunit of the human NoRC (nucleolar remodeling complex), at K633^[50]^. Depletion of KAT8 with short-hairpin RNA in NIH3T3 cells decreases the association of H4K16ac and TIP5 with ribosomal RNA gene (rDNA) and impairs NoRC nucleolar localization, indicating that KAT8-mediated K633 acetylation of TIP5 is essential for NoRC-mediated rDNA silencing. KAT8 also directly interacts with interferon regulatory factor 3 (IRF3) through its catalytic domain and promotes IRF3 acetylation at K359 in macrophages^[51]^. This acetylation inhibits the recruitment of IRF3 to type I interferon (IFN-I) gene promoters, decreasing IRF3 transcriptional activity and antiviral innate immunity. These findings indicate that KAT8 is crucial for cell death, oxidative stress, and inflammation signaling through acetylation of both histone and non-histone proteins.

The *KAT8* gene is ubiquitously expressed across tissues at similar levels^[52]^. KAT8-deficient embryos cannot develop beyond the expanded blastocyst stage and die at implantation with a specific loss of H4K16ac^[53,54]^, indicating that KAT8 is functionally nonredundant with other MYST family of KATs and essential for maintaining H4K16ac and normal chromatin architecture in both male and female embryos. Vav1-Cre-mediated homozygous KAT8 loss in HSCs leads to lethal hematopoietic failure in mice at an early postnatal stage. While KAT8 deletion does not impact fetal hematopoiesis on E14.5, it causes hematopoietic defects on E17.5^[55]^. Similarly, polyinosinic-polycytidylic acid-induced homozygous KAT8 loss in adult mice using the Mx1-Cre system results in dramatic hematopoietic failure. A KAT8-G327E, KAT-activity dead, mutant shows a loss of H4K16ac and reduced survival of adult hematopoietic cells. These findings indicate that KAT8 is essential for the maintenance of hematopoiesis in a developmental stage-specific manner. Additionally, a recent study suggests that KAT8-mediated H4K16ac is a key determinant of HSC heterogeneity, with a CD93^+^ HSC subpopulation increasing in KAT8-low HSCs, which have enhanced proliferative capacity^[56]^. In humans, heterozygous *de novo KAT8* variants in the chromobarrel and catalytic domains are associated with intellectual disability, seizures, autism, and dysmorphisms^[57]^. Cerebrum-specific KAT8 knockout mice, generated with Emx1-Cre, exhibit cerebral hypoplasia in the neocortex and hippocampus, premature neurogenesis, growth retardation, and early lethality before weaning, largely due to improper neural stem and progenitor cell development and massive apoptosis.

### HBO1

HBO1 (histone acetyltransferase binding to ORC1), also known as KAT7 or MYST2, is a member of the MYST family of KATs. Structurally, it contains a MYST-type catalytic domain that includes a zinc finger motif and an acetyl-CoA binding site, both of which are essential for its enzymatic activity^[58]^. HBO1 is primarily localized in the nucleus, where it plays a key role in chromatin modification and the positive regulation of DNA replication [Table 1]. It acetylates histones H3K14, H4K5, H4K8, and H4K12, modifications associated with transcriptional activation and replication origin licensing^[59,60]^. In addition to histones, HBO1 acetylates non-histone substrates, including members of the DNA prereplication complex, such as origin recognition complex (ORC2), minichromosome maintenance complex (MCM2), and cell division cycle 6 (CDC6), affecting DNA replication and cell cycle progression in human cell lines^[58,61]^. However, a recent study challenges some aspects of this role, showing that HBO1 is essential for H3K14ac but not for H4 acetylation, and that it is dispensable for DNA replication and cell proliferation in human cells^[62]^.

Genetic deletion of HBO1 leads to over a 90% reduction in H3K14ac without affecting other histone residues, accompanied by widespread decreases in gene expression. HBO1-deficient embryos exhibit high rates of cell death and DNA fragmentation, resulting in the degeneration of mesenchymal tissues and embryonic lethality^[59]^. In neural stem cells, HBO1 deletion abrogates cellular plasticity, diverse differentiation pathways, and cerebral cortex development^[63]^, highlighting its essential role in H3K14 acetylation, which is indispensable for the activation of repressed genes rather than maintaining transcription in already active genes. These findings indicate that HBO1 prepares chromatin for transcriptional activation. Furthermore, HBO1 is essential for regulating HSC maintenance and self-renewal in adult hematopoiesis^[64]^. Mice with Mx1-Cre-mediated deletion of HBO1 develop hematopoietic failure with pancytopenia in both the blood and bone marrow within 2–6 weeks post-deletion. Additionally, HBO1-mediated H3K14ac is essential for enhancing the expression of the key genes, including *Hoxa9* and *Hoxa10*, necessary for maintaining functional leukemia stem cells^[65]^. A small-molecule HBO1 inhibitor, WM-3835, showed efficacy in targeting primary acute myeloid leukemia cells from patients.

### Subcellular localization of MYST family KATs

The MYST family of KATs is primarily localized in the nucleus [Table 1]; however, some studies suggest that KAT6A, KAT6B, and Tip60 can also locate or translocate into the cytoplasm, as evidenced by immunofluorescence staining and subcellular fractionation analyses in certain cancer cells. However, rigorous experiments are needed to clarify the functional roles of cytoplasmic MYST family KATs. Like other KATs, HBO1 is predominantly localized in the nucleus, where it acetylates 14–3-3 at K49/51, promoting the osteogenic differentiation of human adipose-derived stem cells (ASCs). Interestingly, confocal microscopy suggests the presence of some levels of cytoplasmic HBO1 in ASCs, although its functional significance remains unknown^[66]^. Zou *et al.* demonstrated that while HBO1 primarily functions in the nucleus, its turnover is regulated in the cytoplasm, where Fbxw15 (F-box and WD-40 domain protein 15), a ubiquitin E3 ligase subunit, directly interacts with HBO1 to mediate the ubiquitination and degradation of HBO1^[67]^. This pathway regulates H3K14 acetylation, cell replicative capacity, and endotoxin-induced HBO1 depletion in murine lung epithelial cells. The subcellular localization change of KAT8 is discussed in the Section “CARDIOVASCULAR DISEASE AND ACETYLTRANSFERASES”.

### GNAT (GCN5/PCAF) family

The GNAT (GCN5-related N-acetyltransferase) family consists of three members [Figure 2]: HAT1, KAT2A, and KAT2B. These proteins share a highly conserved GCN5-related N-terminal acetyltransferase domain, which includes an acetyl-CoA-binding motif and a bromodomain. The bromodomain specifically binds to acetylated lysine residues on histone tails.

### HAT1

Human histone acetyltransferase type B catalytic subunit (HAT1), encoded by the *HAT1* gene and also known as KAT1, is a member of the GNAT family of acetyltransferases. It was the first KAT to be discovered^[68]^, initially isolated from *Saccharomyces cerevisiae*^[69]^ with its histone acetyltransferase activity and later from human cells. In humans, HAT1 consists of two polypeptides: the catalytic subunit HAT1 and the regulatory subunit retinoblastoma-binding protein 46 (RbAp46), which forms a complex with HAT1 and stimulates its acetyltransferase activity^[70]^. Structurally, HAT1 contains a catalytic HAT domain responsible for its enzymatic function. Initially recognized for its roles in the cytoplasm as a type B KAT, later studies suggest that HAT1 primarily functions in the nucleus^[71]^, where it di-acetylates newly synthesized histone H4K5 and H4K12, and to a lesser degree, H2A at K5 during chromatin assembly^[72]^ [Table 1]. In addition to its role in H4 acetylation, HAT1 is essential for maintaining newly synthesized H3K9ac, H3K18ac, and H3K27ac during replication-coupled chromatin assembly^[72]^.

HAT1 has been shown to localize in mitochondria and be involved in the acetylation of mitochondrial proteins^[73]^. Acetylproteome analysis using HAT1^+/+^ and HAT1^−/−^ MEFs identified glycolysis-related, chromatin-related, and DNA-binding proteins as the most prominent targets of HAT1-dependent acetylation. Notably, this study also revealed that two acetyltransferases, CBP and HBO1, are acetylated in a HAT1-dependent manner. Additionally, a subset of mitochondrial proteins, including AGK (acylglycerol kinase), ATP5B, and DECR1 (2,4-dienoyl CoA reductase), were identified as targets of HAT1-dependent acetylation. In line with these findings, subcellular fractionation and confocal microscopy analyses further confirmed HAT1 localization in the mitochondria. These observations align with the evidence that HAT1 deletion leads to mitochondrial dysfunction, which is discussed in the Section “AGING AND ACETYLTRANSFERASES”.

Germline HAT1 KO mice show craniofacial defects with abnormalities in skull and jaw bones, and neonatal lethality due to severe defects in lung development. These defects primarily arise from hyperproliferation of mesenchymal cells, leading to severe atelectasis, poor lung aeration, and respiratory distress^[72]^.

### KAT2A

KAT2A, also known as GCN5 (general control nonderepressible 5) or GCN5L2, is a member of the GNAT family of KATs. KAT2A and KAT2B are paralogous genes with ~73% amino acid sequence homology^[74,75]^. Structurally, both KAT2A and KAT2B contain a PCAF homology domain, a bromodomain that binds specifically to acetylated lysine residues, and a catalytic HAT domain responsible for their acetyltransferase activity. KAT2A and KAT2B redundantly acetylate H3K9 and H3K14, regulating chromatin remodeling, DNA replication, DNA repair, and transcription [Table 1]. KAT2A also acetylates non-histone proteins such as c-Myc at K323 and K417 in H1299 cells, dramatically increasing c-Myc stability by tripling its half-life^[37]^. Moreover, E2F1 recruits KAT2A, where KAT2A acetylates H3K9 at the promoter regions of *cyclin E1*, *cyclin D1*, and *E2F1* genes, facilitating the transcription of these genes, which drives A549 cell proliferation *in vitro* and promotes tumorigenesis in mice *in vivo*^[76]^. In addition, KAT2A acetylates PGC-1α in hepatocytes, which inactivates PGC-1α, resulting in dysregulated glucose metabolism^[77]^. The small molecule compound SR-18292 increases the interaction between PGC-1α and KAT2A, suppressing the PGC-1α transcriptional activity, which lowers fasting blood glucose, enhances hepatic insulin sensitivity, and improves glucose homeostasis in high-fat diet-induced diabetic mice^[78]^.

Loss of KAT2A in mouse embryos fails to form dorsal mesoderm lineages, such as chordamesoderm and paraxial mesoderm, due to severe apoptosis, while the cardiac mesoderm remains unaffected. These embryos die during embryogenesis^[79]^. Another study independently demonstrated that KAT2B KO mice develop normally, likely due to functional compensation by KAT2A, whereas KAT2A KO embryos die between days 9.5 and 11.5 of gestation. This difference is likely due to the distinct mRNA expression patterns, with KAT2A expressed at high levels by day 8, and KAT2B first detected on day 12.5^[80]^. These findings suggest that KAT2A and KAT2B have distinct but functionally overlapping roles in embryogenesis. A recent study also showed that simultaneous inactivation of KAT2A and KAT2B in the intestinal epithelium reduces mitochondrial protein acetylation and leads to the accumulation of mitochondrial double-stranded RNA. This activates intrinsic interferon signaling, severely impairing intestinal stem cell renewal and function, ultimately causing lethality within a week after the loss of *KAT2* paralogs^[81]^.

Histone acetylation is linked to memory consolidation in neurons. RNA sequencing of the hippocampal CA1 region from adult mouse brains, a key region for memory function, identified KAT2A as the most highly expressed KAT^[42]^. Genetic deletion of KAT2A specifically in excitatory neurons of the adult forebrain resulted in impaired hippocampus-dependent memory consolidation, alongside disrupted synaptic and nuclear plasticity due to altered NF-κB acetylation and subsequent gene expression changes. In addition to neural function, abnormal histone acetylation is linked to acute myeloid leukemia (AML). KAT2A acetylates CCAAT/enhancer-binding protein alpha (C/EBPα) at K298 and K302 in human myeloid cell lines, reducing the DNA-binding ability of C/EBPα and repressing its transcriptional activity^[82]^. Acetylation of C/EBPα is increased in human AML. Correspondingly, interferon response-inducible *Mx1-Cre*-mediated inhibition of KAT2A in a retroviral-delivered MLL-AF9 model of AML in mice led to the depletion of AML stem-like cells, imposing a mild delay to disease initiation and severely impairing AML propagation, accompanied by a specific loss of H3K9 acetylation at a subset of promoters^[83]^. These findings indicate that KAT2A supports leukemia propagation by maintaining leukemia stem-like cells.

KAT2A is primarily localized in the nucleus; however, it has also been observed in the cytoplasm, where it promotes the acetylation of α-tubulin. This acetylation enhances microtubule polymerization and stability, inhibiting the directional migration of vascular smooth muscle cells^[84]^. This acetylation, along with subsequent microtubule reassembly and stabilization, was suppressed through a direct interaction between KAT2A and LC3 (microtubule-associated protein 1 light chain 3), which facilitates the selective autophagic degradation of KAT2A.

### KAT2B

KAT2B, also known as PCAF [p300/CREB-binding protein (CBP)-associated factor], is a member of the GNAT family of KATs. It specifically acetylates histone H3K9 and H3K14^[85]^. Structurally, KAT2B features a PCAF homology domain, a bromodomain that recognizes acetylated lysine, and a catalytic HAT domain essential for its acetyltransferase activity. KAT2B was first identified through its competitive inhibition by the oncoprotein E1A, which disrupts the interaction between KAT2B and p300/CBP^[74]^. Overexpression of KAT2B counteracts the E1A function, thereby inhibiting cell cycle progression in HeLa cells. Beyond histone acetylation, KAT2B also acetylates p53 at K320 within its nuclear localization signal in response to DNA damage, enhancing the DNA binding capability of p53 to its target genes^[86,87]^. These actions suggest that PCAF might function as a tumor suppressor by modulating p53 activity.

Germline KAT2B KO mice are born near the expected Mendelian ratio^[80]^. Homozygous KAT2B KO mice show no obvious phenotypic abnormalities and reach sexual maturity at a comparable age. This normal phenotype is likely due to compensatory functions from KAT2A. However, later observational studies have shown that KAT2B deletion affects lifelong memory formation and response to stress^[88,89]^. KAT2B KO male and female mice exhibited short-term memory deficits and exaggerated response to acute stress at 2 months old, which were associated with alterations in the organization of the pyramidal cell layer in the hippocampus and activation of MAP kinase^[88]^. These learning and memory impairments became more pronounced at 6 and 12 months of age.

### p300/CBP family

CREB-binding protein (CBP) and its paralog p300 are ubiquitously expressed proteins with similar domain structures that act as scaffolds for interactions with hundreds of transcription factors [Figure 2]. As a result, CBP/p300 serves as a central integrator of multiple nuclear signal transduction pathways by interacting with various transcription factors^[90]^ [Table 1]. Both acetyltransferases share several key interaction and catalytic domains: the HAT domain, the nuclear receptor interaction domain (NRID), the KIX domain - a highly conserved three-helix bundle that interacts with numerous transcription factors, the cysteine/histidine-rich domains [TAZ1(CH1), CH2, and TAZ2(CH3)] that bind cellular regulatory proteins, the bromodomain that recognizes acetyl-lysine residues in histone tails, and the nuclear receptor coactivator binding domain (NCBD). Although CBP and p300 are largely redundant in cells *in vitro*^[91,92]^, they possess unique properties *in vivo*, especially in maintaining normal hematopoiesis, T-cell function, and organ development^[93,94]^. Notably, deletion of either protein or a double heterozygous deletion leads to early embryonic lethality in mice, highlighting their essential roles^[95,96]^.

### CBP

CBP (CREB-binding protein), also known as KAT3A, is a member of the p300/CBP family of KATs. CBP acetylates histones H3K18 and H3K27, modifications crucial for the recruitment of RNA Polymerase II and the activation of nuclear receptor target genes in response to ligands^[97]^. CBP also acetylates numerous non-histone substrates, including transcription factors such as p53 and NF-κB, thereby influencing their activity and stability in diverse cellular processes, including DNA repair, cell growth, and immune responses.

CBP can translocate from the nucleus to the cytoplasm in response to interferon α, enhancing its interaction with and acetylation of Stat1 at K410 and K413. This modification facilitates Stat1 binding to the NF-κB p65 subunit, inhibiting its nuclear localization and suppressing the NF-κB activity^[98]^. Similarly, translocation of CBP or PCAF from the nucleus to the cytoplasm following cellular damage, such as UV exposure, promotes the acetylation of Ku70 at its C-terminus. This disrupts the interaction between Ku70 and Bax, thereby leading to Bax activation and Bax-mediated apoptosis^[99]^.

Mutations in the *Cbp* gene are associated with Rubinstein-Taybi syndrome (RTS), an autosomal dominant disorder, characterized by mental retardation, craniofacial malformations, heart defects, and abnormal pattern formation. Homozygous CBP deletion in mice results in early embryonic lethality around embryonic days 10.5–12.5, while heterozygous CBP KO mice display various skeletal abnormalities^[95,100]^. The lethality in homozygous KO embryos is due to massive hemorrhage caused by defective blood vessel formation in the central nervous system. CBP-deficient embryos also exhibit developmental delays and impaired hematopoiesis without noticeable heart abnormalities, indicating that cardiac anomalies observed in RTS patients may result from a dominant negative effect of mutant CBP. Indeed, heterozygous CBP KO mice expressing truncated CBP protein (residues 1–1084) exhibit RTS-like features, including growth retardation and cardiac and skeletal anomalies^[101]^.

### p300

p300, also known as KAT3B, is a member of the p300/CBP family and functions as a prominent histone acetyltransferase and transcriptional coactivator. p300 acetylates all four core histones within nucleosomes and also targets various non-histone substrates, including p53, NF-κB, c-Myc, and the androgen receptor^[102,103]^. These acetylation events influence crucial cellular processes such as DNA repair, cell cycle regulation, differentiation, and immune responses^[96,104]^.

Homozygous p300 KO embryos die between embryonic days 9 and 11.5, exhibiting defects in neurulation, cell proliferation, and heart development^[96]^. On E10.5, around 20% of homozygous p300 KO embryos show an enlarged heart cavity with no overt patterning defects and poor yolk sac vascularization, along with significantly reduced trabeculation in the ventricular chambers. Furthermore, heterozygous p300 KO mice also exhibit considerable embryonic lethality, and double heterozygosity for p300 and CBP results in early embryonic death.

### b. Non-canonical KATs

We define non-canonical KATs as proteins that are components of transcription coactivator or nuclear receptor coactivator complexes and possess intrinsic acetyltransferase catalytic activity. While the number of identified non-canonical KATs is growing, we will focus on key non-canonical KATs, which are categorized into two primary groups [Figure 2]: transcription coactivator-related KATs and steroid receptor coactivator-related KATs.

### Transcription coactivator-related KATs

Transcription coactivator-related KATs include three acetyltransferases: TAF1 (KAT4), ELP3 (KAT9), and GTF3C4 (KAT12), each forming distinct complexes that regulate transcription [Table 1].

### TAF1 (KAT4)

TAF1 (TATA-binding protein-associated factor 1), also known as TAFII-250, has limited sequence similarity with other KATs. Structurally, it contains a KAT domain, two C-terminal bromodomains, and Ser/Thr kinase domains at both N- and C-terminals, which can autophosphorylate or transphosphorylate other transcription factors^[105–107]^. TAF1 interacts with and phosphorylates p53 at Thr55, promoting p53 degradation and facilitating G1 cell cycle progression^[108]^. The tandem bromodomains of the human TAF1 preferentially bind to a diacetylated over monoacetylated histone H4 tails, aiding in the recruitment of transcription factors and complex formation^[109]^. As the largest and core scaffold subunit, TAF1 forms a transcription factor IID (TFIID) complex with other TAFs (TAF2 to TAF13), which initiates the assembly of the RNA polymerase II (Pol II)-dependent transcription machinery^[110]^.

The *Taf1* gene is located on the X chromosome, and its variants are associated with X-linked dystonia-parkinsonism (XDP), a disorder found exclusively in patients of Filipino ancestry. This condition primarily affects males and is characterized by adult-onset dysmorphism and intellectual disability^[111]^. Integrated genome and transcriptome assembly technologies have revealed that a SINE-VNTR-Alu retrotransposition into intron 32 of the *Taf1* gene is linked to XDP through an aberrant transcriptional mechanism^[112]^. Recent research has demonstrated that phenotypes associated with TAF1 variants exhibit pleiotropy and clinical variability, manifesting as prominent brain morphological abnormalities, seizures, and heart malformations^[113]^. CRISPR-Cas9-mediated editing of the *Taf1* gene in rat pups has revealed that TAF1-edited rats display behavioral deficits during both neonatal and juvenile stages, along with hypoplasia and loss of Purkinje cells^[114]^. In male mice, TAF1 deletion results in embryonic lethality, which explains the absence of reported null variants in humans^[115]^. Interestingly, TAF1 heterozygous KO female mice display no overt brain abnormalities but show significantly increased weight with age and reduced movement, suggesting that specific neuronal populations are adversely affected by TAF1 deletion.

### ELP3 (KAT9)

The transcription elongator complex protein 3 (ELP3) is the catalytic tRNA acetyltransferase subunit of the elongator complex, involved in multiple tRNA modifications, including mcm5U (5-methoxycarbonylmethyl uridine), mcm5s2U (5-methoxycarbonylmethyl-2-thiouridine), and ncm5U (5-carbamoylmethyl uridine). These modifications play an important role in transcription elongation. ELP3 contains a C-terminal KAT domain, which acetylates the N-terminal tails of histone H3 and, to a lesser extent, histone H4^[116]^, as well as non-histone proteins, such as α-tubulin^[117]^. Additionally, ELP3 contains a radical S-adenosyl methionine (rSAM) domain^[118]^. This unique combination of domains and functions categorizes ELP3 as a non-canonical KAT.

Variants of the *Elp3* gene are associated with motor neuron degeneration and amyotrophic lateral sclerosis (ALS)^[119]^. ELP3 expression is reduced in the motor cortex of ALS patients and correlates with mcm5s2U levels. Ubiquitous, but not neuron-specific, overexpression of ELP3 attenuated neurodegeneration in the SOD1^G93A^ mouse model of ALS^[120]^. The protective effects of ELP3 are mediated by the SAM domain, rather than the KAT domain, as confirmed in ALS zebrafish models^[120]^. Homozygous deletion of ELP3 in mice is lethal around embryonic day 8, highlighting its critical role in mammalian embryogenesis^[120]^. The Elongator complex, which includes Elp3 and Elp1, is also involved in the maturation of projection neurons^[117]^. The ELP3-enriched fraction acetylates α-Tubulin, promoting radial migration and branching of cortical projection neurons.

### GTF3C4 (KAT12)

General transcription factor 3C polypeptide 4 (GTF3C4), also known as KAT12 or the transcription factor IIIC 90 kDa subunit (TFIIIC90), is a component of the transcription factor IIIC2 (TFIIIC2) complex, which comprises six subunits: GTF3C1, GTF3C2, GTF3C3, GTF3C4, GTF3C5, and GTF3C6^[121]^. The *GTF3C4* gene is located on Chromosome 9 in humans^[122,123]^. *In vitro* HAT assays have shown that GTF3C4 possesses histone acetyltransferase activity with a preference for histone H3 as its substrate^[124]^. The TFIIIC complex recognizes promoter elements and facilitates the recruitment of TFIIIB and RNA polymerase III, thereby initiating transcription.

### Steroid receptor coactivator-related KATs

The p160 steroid receptor coactivator (SRC) family, comprising three homologous members (SRC1, SRC2, and SRC3), plays a key role in mediating the transcriptional function of nuclear receptors (NRs) and other transcription factors^[125,126]^. SRCs contain several conserved regions, including the basic helix-loop-helix (bHLH) and Per/Arnt/Sim (PAS) domains (b-HLH-PAS), a central NR-interacting domain, and two C-terminal activation domains, AD1 and AD2. These activation domains function as strong mediators of epigenetic enzymatic activities and exhibit intrinsic acetyltransferase activity necessary for gene transcription regulation^[127]^. While the exact role of SRC acetyltransferase activity in the heart remains under investigation, we will provide a brief introduction to steroid receptor coactivator-related KATs in this section.

### Nuclear receptor coactivator 1

Nuclear receptor coactivator 1 (NCOA1), also known as KAT13A or SRC1, is a nuclear receptor coactivator that directly binds nuclear receptors or transcription factors to enhance transcriptional activity in a hormone-dependent manner. Discovered in 1995 as the first member of the SRC family^[128]^, NCOA1 (SRC1) exhibits intrinsic KAT activity, specifically targeting histone H3 and H4^[129]^.

### Nuclear receptor coactivator 2

Nuclear receptor coactivator 2 (NCOA2), also referred to as transcriptional intermediary factor 2 (TIF2), KAT13C, or SRC2, is a nuclear receptor coactivator that acts as a scaffold for transcription factors in a hormone-dependent manner. SRC2 (TIF2) was the second identified member of the SRC family, cloned in 1996^[130]^. TIF2 KO mice are viable, but both sexes exhibit impaired fertility and reduced expression of fasting-induced glucose-6-phosphatase (G6Pase), indicating that TIF2 plays a critical role in mouse reproductive functions, energy production, and gluconeogenesis^[131,132]^. In line with the crucial role of SRCs in regulating energy metabolism in part through peroxisome proliferator-activated receptors, germline TIF2 KO mice are resistant to high-fat diet-induced obesity with enhanced adaptive thermogenesis, whereas SRC1 KO mice are more susceptible to obesity due to decreased energy expenditure^[133]^.

### Nuclear receptor coactivator 3

Nuclear receptor coactivator 3 (NCOA3), also known as ACTR, AIB-1 (amplified in breast cancer 1 protein), SRC3, KAT13B, pCIP (CBP-interacting protein), RAC3 (receptor-associated coactivator 3), or TRAM-1 (thyroid hormone receptor activator molecule 1), is a nuclear receptor coactivator that directly interacts with nuclear receptors and stimulates their transcriptional activities in a hormone-dependent fashion. AIB-1 was originally amplified from the human breast cancer cell line BT-474 and was named AIB-1^[134]^. It was subsequently identified as a coactivator by several independent groups and later termed SRC3. NCOA3 also acetylates both free and nucleosome histones with a substrate preference for histones H3 and H4^[135]^.

### Circadian locomoter output cycles protein (KAT13D)

Circadian locomoter output cycles protein (CLOCK), also referred to as class E basic helix-loop-helix protein 8 (bHLHe8), is a transcriptional activator that serves as a core component of the circadian clock, an internal time-keeping system. The transcription and translation of core clock components, composed of CLOCK, NPAS2, BMAL1, BMAL2, PER1, PER2, PER3, CRY1, and CRY2, play a central role in circadian rhythm generation. CLOCK possesses intrinsic acetyltransferase activity, similar to the acetyl-coenzyme A binding motifs found in the MYST family of KATs, and acetylates histone, specifically H3K9 and H3K14^[136]^, as well as non-histone proteins, including its partner BMAL1^[136]^. The acetylation of BMAL1 by CLOCK facilitates the recruitment of CRY1 to the CLOCK-BMAL1 complex, thereby promoting transcriptional repression^[137]^.

### c. Sialic acid O-acetyltransferases (SOATs)

*O*-acetylation of sialic acids, nine-carbon monosaccharides located at the termini of glycans on the cell surfaces^[138]^, occurs at the C-4/7/8/9 positions and is among the most common and critical PTMs of sialic acids^[139]^. This modification was first identified by mass spectrometry in 1975^[140]^. Glycans, composed of multiple monosaccharides linked by glycosidic bonds, can exist as free molecules or be attached to proteins (glycoproteins) or lipids (glycolipids). They are crucial for biological processes, including cell-cell communication, protein folding and stability, host-pathogen interaction, and signaling, in particular immune regulation. Consequently, aberrant modifications in their terminal sialic acids contribute to the pathogenesis of various human diseases. Clinical studies have demonstrated that circulating levels of *O*-acetylated glycans are closely linked to inflammation, cancer, and autoimmune diseases, as discussed in a later section.

*O*-acetylation occurs in the Golgi system, where glycoproteins or glycolipids, particularly sialic acid, undergo *O*-acetylation mediated by sialic acid *O*-acetyltransferases (SOATs) and *O*-acetylesterases (SIAEs). These enzymes add or remove an acetyl group at one or more of the hydroxyl groups at the C-4, C-7, C-8, or C-9 positions, respectively^[141]^. Functionally defective rare and polymorphic variants of SIAE are strongly linked to increased susceptibility to autoimmune diseases, including rheumatoid arthritis and type I diabetes^[142]^. Although this reaction takes place on sugar residues rather than amino acids or fatty acids, we will briefly discuss SOATs due to their potential clinical relevance.

### CAS1 domain containing 1 (CASD1)

In mammals, only a single SOAT has been identified to date: capsule structure (CAS) 1 domain-containing 1 (CASD1)^[143,144]^. Human CASD1 is a multimembrane-spanning protein of 797 amino acids localized in the Golgi system. Homology modeling predicts that residues 83–290 form the catalytic domain of CASD1, which exhibits a preference for CMP-sialic acids as substrates. CRISPR/Cas-mediated CASD1 deletion completely abolished *O*-acetylation in cells^[144]^. Furthermore, *in vitro* acetyltransferase activity assays using the purified N-terminal luminal domain of CASD1 demonstrate the transfer of acetyl groups from acetyl-CoA to CMP-activated sialic acid, providing direct evidence that CASD1 functions as a SOAT^[144]^.

### d. N-terminal acetyltransferases

N-terminal acetyltransferases (NATs) co-translationally transfer an acetyl group from acetyl-CoA to the α-amino group of a protein or polypeptide N-terminus, introducing charge neutralization to the N terminus, which affects protein stability, folding, complex formation, and subcellular localization. A proteomics analysis using 742 human protein N termini revealed that approximately 84% of human proteins undergo N-terminal acetylation^[145]^. NATs structurally share the catalytically active GNAT fold, an evolutionarily conserved feature of NATs, positioning NATs as a subfamily of the GNAT superfamily. However, NATs differ from GNAT family KATs by two key features: (1) The peptide binding site in NATs is usually flanked by the β6-β7 and α1-α2 loops, which are directly involved in N-terminal substrate binding and adopt a relatively closed conformation, thus reducing affinity for lysine side chain binding; and (2) NATs typically form extensive hydrogen bonds with the backbone atoms of the first 2–3 N-terminal residues, further enhancing their specificity for protein N-termini^[146]^.

Seven NATs have been identified in humans, each with distinct functions, subcellular localization, composition, and substrate specificity. The detailed function and structures of NATs have been recently reviewed elsewhere^[146,147]^; we will briefly introduce NATs to discuss the latest understanding of their roles in cardiometabolic disease and aging in a later section. Unlike lysine acetylation, which is reversible and mediated by KATs, no deacetylase has been identified for reversing NAT-mediated N-terminal acetylation^[147]^. Regarding substrate specificity, the first two amino acids at the N-terminus of a protein are crucial in determining whether it is acetylated and which NAT catalyzes the process. For example, serine at the N-terminus is frequently acetylated, whereas glycine and proline are rarely acetylated. In terms of subcellular localization, NatA to NatE are cytoplasmic and ribosome-associated, NatF is located on the Golgi membrane, NatG in the organelle lumen, and NatH in the cytoplasm but not ribosome-associated. Despite the high prevalence of N-terminal acetylation in eukaryotic proteins, the precise cellular roles of N-terminal acetylation remain largely unknown.

### NatA (N-terminal acetyltransferase A)

NatA is the primary NAT, responsible for acetylating approximately 40% of the human proteome in a co-translational manner^[148,149]^. NatA is composed of the catalytic subunit Naa10 and the auxiliary subunit Naa15, which acts as a ribosomal anchor and provides the substrate specificity, along with the Huntingtin yeast two-hybrid protein (HYPK), which acts as a negative regulator of NatA activity^[150–152]^. Additionally, another catalytic subunit, Naa50, can interact with Naa15 to form a multienzyme complex known as NatA/E. During early translation, the nascent polypeptide-associated complex (NAC) assembles a multienzyme complex with NatA and methionine aminopeptidase (MetAP), facilitating the release of the inhibitory protein HYPK, thereby activating NatA on the ribosome for co-translational N-terminal acetylation^[153]^. NatA specifically acetylates N-termini with Ser, Ala, Thr, Gly, Val, and Cys residues after the initiator methionine is removed by MetAP.

### NatB (N-terminal acetyltransferase B)

NatB acetylates approximately 21% of human proteins^[148]^, including actin, tropomyosin, CDK2, and α-synuclein, a key protein implicated in the pathogenesis of Parkinson’s disease. The stability of these proteins is regulated by NatB-mediated N-terminal acetylation^[154,155]^. NatB is composed of the catalytic subunit Naa20 (also known as Nat3p) and the auxiliary subunit Naa25 (Mdm20p), specifically acetylating proteins or peptides with N-termini that start with Met followed by Asp, Glu, Asn, or Gln. The substrate specificity of NatB is determined by hydrogen bonds between the substrate Asp and pocket residues of Naa20^[156]^.

### NatC (N-terminal acetyltransferase C)

The NatC is composed of the catalytic subunit Naa30 and two auxiliary subunits Naa35 and Naa38. The NatC complex co-translationally acetylates a wide range of protein or peptide N-termini that start with a methionine followed by hydrophobic amino acids^[157,158]^. Recent studies revealed the structure of the NatC complex, which showed a catalytic cleft suitable for the acetylation of methionine and accompanying hydrophobic N-termini^[159,160]^. Positional proteomics combined with siRNA-mediated knockdown of hNaa30 in A-431 cells revealed that protein expression in several organelles, particularly in mitochondria, is reduced, with some proteins identified as NatC substrates^[158]^. The siRNA-mediated knockdown of Naa30 disrupted mitochondrial membrane potential and caused mitochondrial fragmentation, suggesting that NatC-mediated N-terminal acetylation is crucial for maintaining mitochondrial integrity and function.

### NatD (N-terminal acetyltransferase D)

NatD is a ribosomal NAT that consists solely of the catalytic unit Naa40, lacking an anchor subunit^[161,162]^. NatD specifically acetylates the N-termini of histones H2A and H4, which in humans contain the sequence Ser-Gly-Arg-Gly^[161]^. MetAP first removes the N-terminal methionine, similar to the process observed with NatA, after which the exposed N-terminal serine residue is acetylated by NatD.

### NatE (N-terminal acetyltransferase E)

NatE refers to a complex that contains the catalytic subunit Naa50, which can also interact with the catalytic subunit Naa10 to form a multienzyme complex (NatA/E), along with the auxiliary subunit Naa15 and the inhibitory subunit HYPK^[163]^. NatE co-translationally acetylates protein N-termini^[164]^, influencing protein stability, localization, and interactions. Its substrate specificity overlaps with that of NatC. Human monomeric Naa50 remains stable even in the absence of the NatA complex and primarily acetylates N-terminal Met residues.

### NatF (N-terminal acetyltransferase F)

NatF (Naa60) is solely composed of the catalytic unit Naa60. Unlike the ribosome-associated NATs (NatA to NatE), NatF is uniquely located on the cytosolic side of Golgi membranes, where it post-translationally acetylates the N-termini of various transmembrane proteins^[165,166]^. NatF features a distinct membrane-binding motif at its C-terminus, enabling its association with the Golgi membrane. NatF specifically targets N-terminal Met residues followed by Leu, Ile, Phe, Tyr, and Lys residues.

### NatH (N-terminal acetyltransferase H)

NatH, also known as N-acetyltransferase 80 (Naa80), Fus-2, or NAT6, was initially identified by a computerized sequence homology search^[167]^. NatH is composed solely of the catalytic unit Naa80 and is neither ribosome-associated nor organelle-localized. It is expressed diffusely in the cytosol, where it post-translationally acetylates the N-termini of proteins^[168]^. NatH specifically acetylates the N-terminus of various forms of actin, one of the most abundant cytosolic proteins, which plays a crucial role in cytoskeletal assembly and cell motility. This includes roles in formin-mediated elongation, increasing the ratio of filamentous to globular actin, promoting filopodia, and accelerating cell motility^[168,169]^.

## EFFECT OF DIETS AND DIETARY PATTERNS ON ACETYLTRANSFERASES AND ACETYLATION

Growing evidence indicates the impact of diets on acetyltransferase activity, affecting metabolic pathways and cellular stress responses. This section explores the influence of diets and gut microbiota-derived metabolites on metabolic changes and post-translational modifications regulated by acetyltransferases [Figure 1].

Dietary unsaturated fatty acids, abundant in the Mediterranean diet, are associated with a lower risk of inflammation and cardiovascular disease and increased longevity^[170]^, partly through the regulation of lipid metabolism and reduction of lipotoxicity^[3,171]^. Circulating levels of glycoprotein acetylation serve as markers of inflammation, alongside high-sensitivity C-reactive protein (hs-CRP) and interleukin-6 (IL-6). Sialic acid *O*-acetylation, involved in various biological and disease processes, can be measured as glycoprotein acetylation using nuclear magnetic resonance spectroscopy. A cohort study of 25,315 initially healthy U.S. women found that greater adherence to the Mediterranean diet was linked to lower circulating glycoprotein acetylation levels. Decreased inflammatory markers, including glycoprotein acetylation, were major contributors to the reduced risk of mortality and diabetes onset associated with the Mediterranean diet^[172–174]^. In addition, higher dietary magnesium intake is associated with lower inflammation, with glycoprotein acetylation levels, hs-CRP levels, and leukocyte counts inversely correlated with dietary magnesium intake^[175]^.

Although glycoprotein acetylation can be a marker of inflammation, its levels and those of hs-CRP are influenced by distinct biological pathways. The ELSA-Brasil cohort study of 15,105 civil servants revealed that higher CRP levels are primarily associated with obesity, whereas elevated glycoprotein acetylation is linked to a greater burden of cardiovascular risk factors and an increased likelihood of carotid artery plaque^[176]^. Consistent with these findings, hs-CRP levels are associated with body mass index without a direct link to cardiometabolic phenotypes in adolescents and young adults, whereas glycoprotein acetylation is associated with adverse cardiometabolic risk profiles starting in adolescence and predicts future risk of developing cardiometabolic disease^[177]^. These findings underscore the association between glycoprotein acetylation and cardiometabolic disease, though it remains unclear whether glycoprotein acetylation is merely a surrogate marker of inflammation or whether acetyltransferases involved in this process, such as CASD1, play a direct role in the development of cardiometabolic disease.

### Macronutrients, gut microbiota-derived metabolites, and acetylation

Acetyltransferases play a critical role in cellular metabolism. siRNA-mediated knockdown of KAT8, GCN5, NAA40, or NAA10 has been shown to enhance lipid synthesis by increasing intracellular acetyl-CoA levels without altering the expression of lipid synthesis-related genes in AML12 hepatocytes, as demonstrated by HPLC-MS and GC-MS analyses^[178]^. A recent study showed that amino acid or glucose depletion induces the translocation of p300 from the cytoplasm to the nucleus, decreasing acetylation of the mTORC1 component raptor, which reduces mTORC1 activity and enhances autophagy in most cell types^[179]^. Conversely, leucine and its metabolite acetyl-CoA inhibit autophagy by promoting p300-dependent acetylation of raptor at K1097, resulting in the activation of mTORC1^[180,181]^.

Metabolic and environmental changes can influence post-translational modifications, including various histone acylations, such as acetylation, butyrylation, crotonylation, and propionylation^[182]^, through gut microbiota-derived metabolite changes. The gut microbiome serves as a central hub connecting diets, dietary components, and cardiometabolic health^[3,183,184]^. For instance, gut microbiota-derived short-chain fatty acid (SCFA) butyrate enhances histone H3 crotonylation at lysine 18 by inhibiting histone deacetylase activity in the mouse colon, which is linked to cell cycle progression. Conversely, the reduction of gut bacteria and SCFAs in the colon lumen and serum using a cocktail of antibiotics leads to a noticeable global decrease in histone crotonylation in the colon^[185]^. Additionally, microbiota-derived SCFAs have been shown to enhance acetylation, butyrylation, and propionylation of histone H3 at K9 and K27 in the intestinal epithelial cells of female mice^[186]^. These findings clearly illustrate how diets and environmental factors regulate histone modifications through gut microbiota-derived metabolites. Further investigations are warranted to determine how diet-induced histone acylations in the colon influence cardiovascular disease and aging.

### Ketone metabolism and acetylation

The heart primarily utilizes fatty acids as its main fuel source (> 70%) and only a small amount of glucose, as shown by catheter-based metabolomics analyses^[187]^. However, cardiac-specific deletion of the mitochondrial pyruvate carrier (MPC), which imports glucose and lactate metabolites into the mitochondria, results in age-dependent cardiac hypertrophy, a condition characterized by an increase in heart and individual cardiomyocyte sizes^[188]^, and cardiomyopathy in mice^[189,190]^. A prospective cohort study and meta-analysis also showed that both low (< 40% of energy from carbohydrates) and high (>70% of energy from carbohydrates) intake are associated with increased mortality^[191]^, highlighting the critical role of pyruvate metabolism in maintaining heart function. Ketone bodies contribute approximately 15% of myocardial carbon uptake in intact hearts, and failing hearts increasingly rely on ketone bodies as an energy source^[187]^. High-fat, low-carbohydrate ketogenic diets extend longevity, enhance healthspan, and improve memory in adult mice^[192,193]^, potentially due to ketone bodies^[194,195]^. Ketone body supplementation via a ketogenic diet attenuated cardiac hypertrophy and heart failure in mice under acute pressure overload^[196]^, whereas inhibiting the ketone utilization pathway by deletion of the *SCOT* gene exacerbated pathological remodeling in mice^[197]^. Additionally, administration of 3-hydroxybutyrate or ketone esters has shown favorable hemodynamic effects in patients with heart failure with reduced ejection fraction^[198]^ or with cardiogenic shock^[199]^. These cardioprotective effects may be mediated in part by antioxidative effects through class I histone deacetylates^[200]^ and anti-inflammatory effects^[201]^. Beyond the heart, β-hydroxybutyrate prevents vascular senescence^[202]^. In vascular cells, heterogeneous nuclear ribonucleoprotein A1 (hnRNP A1) directly binds to β-hydroxybutyrate, which enhances the mRNA stability of Octamer-binding transcriptional factor 4 (Oct4), increasing the expression of Lamin B1 and reducing DNA damage-induced senescence.

Importantly, both human and mouse failing hearts exhibit mitochondrial protein hyperacetylation^[203]^, and this acetylation is further elevated by ketogenic diets or ketone bodies in the heart^[196,200]^, despite their known cardioprotective effects. These seemingly contradictory observations are also reflected in experimental data. Acetylation of muscle creatine kinase reduced its enzymatic activity and deacetylation through overexpression of sirtuin 2 restored creatine kinase activity by reducing its acetylation^[204]^. Furthermore, interestingly, increasing β-hydroxybutyrate levels has been shown to reduce mitochondrial acetylation and suppress NLRR3 inflammasome formation in a HFpEF mouse model^[205]^. Although the direct relationship between acetylation levels and ketone supplementation in this experiment is not fully demonstrated, a mouse model with hyperacetylation, created by double knockout of carnitine acetyltransferase and sirtuin 3, showed that hyperacetylation of the cardiac mitochondrial proteome has minimal impact on mitochondrial function or the susceptibility of mice to stress-induced heart failure^[206]^. These findings underscore the need for further investigation into the effects of site-specific acetylation on individual proteins, as well as mitochondrial and cardiac function, and to determine whether and how acetyltransferases regulate mitochondrial protein acetylation in response to a ketogenic diet or ketone bodies.

### Relationship between deacetylases and acetyltransferases in acetylation events

The status of acetylation in cells is regulated by enzymatic reactions, which depend on the balance between acetyltransferases and deacetylases, as well as non-enzymatic acetylation directly induced by acetyl-CoA. This highlights that acetyltransferases and deacetylases functionally - and, in some cases, physically - interact with one another based on the cellular levels of acetyl-CoA and NAD^+^. The levels of these coenzymes and cofactors are interdependently modulated by various factors, including diets, environmental conditions, physical activity, and the presence of diseases [Figure 1].

For example, fatty acid palmitate increases acetyl-CoA levels by enhancing mitochondrial fatty acid β-oxidation, which is accompanied by a decrease in NAD^+^ levels^[207]^. A reduction in the NAD^+^/NADH ratio suppresses Sirt1 activity in β cells, leading to insulin resistance, which can be ameliorated with nicotinamide mononucleotide (NMN) treatment by replenishing NAD^+^ levels^[208]^. Similarly, elevated acetyl-CoA levels resulting from excess saturated fatty acids promote Tip60-mediated acetylation of Rheb at K8, which triggers mTORC1 activation, IRS1 serine phosphorylation, and insulin resistance^[207]^. Furthermore, the NAD^+^-dependent deacetylase Sirt2 deacetylates lysine residues in the catalytic domain of the acetyltransferase p300, which is known to undergo autoacetylation^[209]^. These findings underscore the intricate functional and physical interactions between NAD^+^-mediated deacetylases and acetyl-CoA-mediated acetyltransferases in maintaining acetylome homeostasis and cellular metabolism.

Autophagy is a lysosome-mediated degradation process that recycles cellular components (e.g., proteins and lipids), maintains cellular metabolism, and eliminates damaged organelles, playing a pivotal role in cardiometabolic health, aging, and various pathological conditions. Autophagy is tightly regulated by nutritional and energy status, where acetylation plays a critical role in modulating the activities of enzymes and transcriptional factors involved in autophagy initiation and autophagosome formation. This regulation targets core autophagy machinery proteins, such as the ULK1 and Beclin1 complexes, as well as histones and transcription factors like TFEB. For instance, nutrient starvation induces rapid depletion of acetyl-CoA, reducing p300 activity, whereas p300 is required for autophagy suppression under high acetyl-CoA levels^[210]^. Additionally, deprivation of growth factors promotes GSK-3β activation, which phosphorylates Tip60 at Ser86, stimulating its activity. Phosphorylated Tip60, in turn, directly acetylates ULK1 at K162 and K606, thereby promoting autophagy initiation^[211]^. Furthermore, GSK-3β-mediated activation of Tip60 facilitates its interaction with and acetylation of Pacer at K483 and K573, which promotes autophagosome maturation by enhancing its interaction with the HOPS complex and Stx17, as well as influencing cellular lipid metabolism^[212]^.

Beclin1 is acetylated at K430 and K437 by p300 under nutrient-rich conditions, which inhibits autophagosome maturation by promoting its interaction with Rubicon. In response to starvation, Beclin1 is deacetylated by Sirt1, thereby stimulating autophagy^[213]^. Similarly, LC3 is deacetylated at K49 and K51 by Sirt1 during starvation, enabling its interaction with Atg7 and facilitating autophagy^[214]^. Several autophagy-related proteins (ATGs), such as ATG5, ATG7, and ATG12, are also acetylated by acetyltransferases, including p300 and GCN5, and are deacetylated by sirtuins. These findings highlight the essential roles of acetyltransferase-mediated acetylation and deacetylase-mediated deacetylation in regulating autophagy in response to nutritional status, emphasizing their importance in maintaining cellular homeostasis and metabolic balance.

### Exercise and acetylation

Physical activity, particularly aerobic exercise, is well established as a primary and secondary preventive measure for cardiometabolic diseases. Prolonged exercise induces physiological ketosis, which increases NAD^+^ levels and activates NAD^+^-dependent deacetylases. In contrast, a sedentary lifestyle, obesity, and aging decrease the NAD^+^ pool while increasing the acetyl-CoA pool, thereby enhancing acetylation levels, especially in mitochondrial proteins. This leads to alterations in substrate utilization and energy metabolism. Consequently, it is conceivable that while exercise may inhibit acetyltransferase activities, comorbid conditions such as obesity can counteract its beneficial effects on cardiometabolic health. However, whether - and if so, how and which - acetyltransferases mediate the effects of exercise on cardiovascular disease remains largely unexplored.

## CARDIOVASCULAR DISEASE AND ACETYLTRANSFERASES

Acetyltransferases play a crucial role in heart development, and pathogenic variants in these enzymes are linked to congenital heart diseases. For example, mutations in the *KAT6A* gene cause KAT6A syndrome, characterized by intellectual disability, microcephaly, and cardiac defects^[15,215]^. Variants in KAT6B are associated with Say-Barber-Biesecker-Young-Simpson syndrome (SBBYSS) and genitopatellar syndrome (GPS), with about half of the patients exhibiting congenital heart defects, mainly atrial or ventricular septal defects and patent foramen ovale^[216]^. *KAT8* variants are linked to Li- Ghorbani-Weisz-Hubshman syndrome (LIGOWS), which presents heart anomalies in approximately half of the patients^[57]^. In addition, haploinsufficiency of the *KAT8 regulatory NSL complex subunit 1* (*KANSL1*) causes Koolen-de Vries syndrome (KdVS), which includes intellectual disability, heart failure, and hypotonia, partly due to impaired recruitment of the BET protein BRD4 to the NSL complex through reduced H4K16ac^[217]^. Another study identified impaired autophagosome-lysosome fusion as a key pathogenic mechanism in KdVS^[218]^. In line with these findings and previously reported study showing the role of KAT8-mediated H4K16ac in regulating the transcription of autophagy-related genes^[219]^, TGF-β downregulates KAT8, thereby activating autophagy in fibroblasts of systemic sclerosis, which leads to fibroblast-to-myofibroblast transition, collagen release and tissue fibrosis^[220]^.

KAT2A is known to promote gluconeogenesis in the liver^[221]^ and is upregulated in cardiac stromal cells isolated from patients with arrhythmogenic cardiomyopathy^[222]^. Short hairpin RNA-mediated knockdown of KAT2A or pharmacological inhibition of KAT2A with MB-3 significantly reduced intracellular lipid accumulation in those cells, which is a recognized pathological feature of arrhythmogenic cardiomyopathy. In addition, as mentioned earlier, mutations in the *Cbp* and *p300* genes are linked to Rubinstein-Taybi syndrome (RTS) and RTS2, respectively, both characterized by intellectual disability, craniofacial malformations, and heart defects. The impact of abnormal expression of CBP and p300 on cardiovascular disease has been extensively studied and reviewed elsewhere^[223]^, so it will not be further discussed in detail in this section.

Congenital heart disease (CHD) associated with *de novo* heterozygous loss of function variants in Naa10 and Naa15 has been previously reported^[224–227]^. Mechanistic studies using human isogenic induced pluripotent stem cells (iPSCs) derived from CHD patients with Naa15 variants (Naa15+/− iPSC-derived cardiomyocytes) revealed altered expression levels in 562 out of 650 NatA-targeted proteins, suggesting a role of NatA-mediated N-terminal acetylation in protein stability^[228]^. Notably, Naa15+/− iPSC-derived cardiomyocytes exhibited altered levels of 18 out of 81 identified ribosomal proteins, disrupting the interaction of Naa15 with the ribosome and affecting protein expressions, which likely contributes to the cardiac pathology observed in CHD patients with Naa15 variants.

### Vascular disease associated with acetyltransferases

RNA sequencing analysis identified upregulation of the *ALDH1A3* (*aldehyde dehydrogenase family 1 member 3*) gene in pulmonary artery smooth muscle cells (PASMCs) from patients with idiopathic pulmonary hypertension (PAH)^[229]^. ALDH1A3 provides acetyl-CoA, which facilitates KAT2B-mediated H3K27ac at NFYA transcription factor binding sites, leading to increased transcription of cell cycle and glycolytic metabolic genes in PAH PASMCs. Additionally, a clinical observational study found elevated expression levels of canonical KATs, as well as histone acetylation marks H3K9ac, H3K18ac, and H3K14ac, in tissues from human abdominal aortic aneurysm (AAA)^[230]^. Furthermore, significant correlations were observed between localization of KAT2B and endothelial cells, KAT3B and T cells and macrophages, and KAT6A and endothelial and smooth muscle cells in AAA. Another study also reported decreased expression of activating transcription factor 3 (ATF3) in AAA tissues^[231]^. NF-κB recruits the p300 complex to ATF3 promoter regions, enhancing ATF3 expression through H3K27ac, which acts to suppress AAA development. These findings highlight the crucial roles of KATs in the pathogenesis of vascular diseases.

### Ischemic heart disease associated with acetyltransferases

Inhibition of Tip60 specifically in cardiomyocytes using tamoxifen-induced Myh6-Cre recombinase three days after myocardial infarction (MI) attenuated post-infarct apoptosis, scar formation, and cardiac remodeling in mice^[232]^. Additionally, both KAT2A and KAT2B are implicated in the cardiac pathology of ischemic heart disease. The impact of various carbon sources (2C, 3C, 6C, 8C, and 9C) on heart function after myocardial ischemia-reperfusion was assessed in rats^[233]^. Among them, sodium octanoate (8C), a medium-chain fatty acid, was identified as the most effective carbon source for mitigating ischemic injury in rat hearts. Sodium octanoate boosts acetyl-CoA production through medium-chain acyl-CoA dehydrogenase (MCAD), enhancing KAT2A-mediated H3K9ac and stimulating the expression of antioxidant-related genes, which helps protect the heart from MI^[233]^.

Contrary to the protective effect of KAT2A-mediated acetylation, KAT2B exacerbates MI^[234]^. KAT2B protein levels were significantly increased in post-MI myocardium^[234]^. Pharmacological inhibition of KAT2B using Embelin attenuated MI-induced cardiac remodeling and dysfunction in mice. Furthermore, aerobic training suppressed the MI-induced upregulation of KAT2B, contributing to aerobic training-mediated cardioprotection against MI. In line with this finding, KAT2B also plays a role in atherosclerosis progression^[235]^. KAT2B acetylates FoxP3, a critical factor for regulatory T-cell (Treg) differentiation and immune modulation. Genetic inhibition of KAT2B decreased FoxP3+ Treg numbers and ameliorated atherosclerotic lesions in ApoE3Leiden mice fed a high-fat diet, without affecting systemic inflammation.

A recent study demonstrated that CBP acetylates Yes-associated protein (YAP), a key effector of the Hippo signaling pathway, at K265 in cardiomyocytes in the heart in response to MI. This acetylation promotes YAP cytoplasmic localization by facilitating its interaction with TUBA4A, a component of the microtubule network^[236]^. In contrast, YAP-K265R, an acetylation-resistant mutant, led to improved cardiac regenerative capacity in mice, marked by increased nuclear localization of YAP in the heart.

NCOA1–3 (SRC1–3) have been implicated in heart development and are associated with noncompaction cardiomyopathy^[237]^ and pressure overload-induced angiogenesis in mice^[238]^. NCOA1–3 proteins are highly expressed in cardiac fibroblasts in adult mice^[239]^. Activation of NCOA1–3 using the small molecule stimulator MCB-613 reduced fibroblast differentiation and activation, fibrosis, and adverse cardiac remodeling by coactivating the NRF2 antioxidative stress signaling pathway in response to MI in mice^[239,240]^. Collectively, these findings highlight the crucial roles of acetyltransferase-mediated acetylation of histone and non-histone proteins in modulating ischemic heart disease.

### Cardiac mitochondrial function associated with acetyltransferases

KAT8 localizes in the nucleus and mitochondria^[241]^, where it plays a dual role in regulating gene expression. In mitochondria, KAT8 binds to mtDNA through KANSL3, stimulating the expression of mitochondrial genes involved in oxidative phosphorylation, with KAT8 catalytic activity being essential for this function^[241]^. Deletion of KAT8 in the heart and skeletal muscle using muscle creatine kinase (MCK) promoter-driven Cre recombinase leads to mitochondrial degeneration and dysfunction of oxidative phosphorylation in cardiomyocytes, resulting in hypertrophic cardiomyopathy and heart failure in mice. These findings indicate that KAT8 serves as a dual-transcriptional regulator of both nuclear and mitochondrial genomes.

Consistent with these findings, KAT8 and H4K16ac are required for regulating mitochondrial and ciliary gene expression in the skin^[242]^. Epithelial-specific KAT8 KO mice with *Krt14-Cre* exhibit lethality within hours after birth, showing a gradual loss of H4K16ac in the embryonic epidermis and severe defects of the skin, indicating that KAT8 is required for embryonic skin development. Single-cell RNA sequencing revealed widespread downregulation of nuclear-encoded mitochondrial genes in epithelial cell clusters. Furthermore, bulk RNA sequencing analysis using FACS-purified epithelial cells showed significant downregulation of primary cilia-related gene sets and ciliary genes.

Another recent study highlights the crucial role of KAT8 in energy metabolism through mitochondrial protein acetylation^[243]^. Loss of either KAT8 or KANSL complex members disrupts mitochondrial morphology and energy metabolism by reducing COX17 acetylation, independently of the KAT8’s transcriptional regulatory role in MEFs. The KAT8-KANSL complex acetylates COX17 at K18 and K30, maintaining complex IV integrity and mitochondrial cytochrome c oxidase activity in MEFs^[243]^. Collectively, these findings underscore the critical role of KAT8 in maintaining mitochondrial morphology and function, with its disruption leading to mitochondrial and cardiac dysfunction through both epigenetic-dependent and -independent mechanisms. In contrast to these findings, Hu *et al.* recently demonstrated that KAT8 is increased in mitochondria in the hearts of patients with heart failure and mice subjected to pressure overload^[244]^. AAV9-mediated upregulation of mitochondrial KAT8 in cardiomyocytes under the control of the cardiac troponin T promoter was sufficient to induce heart failure in mice. This effect was exacerbated by muscle-specific knockout of Sirt3, highlighting the crucial role of mitochondrial protein acetylation by KAT8 in heart failure progression. Overexpression of mitochondrial KAT8 increased acetylation of ATP5B at K201 in HEK293T cells, as shown by mitochondrial protein isolations followed by tandem mass tag (TMT)-based liquid chromatography and tandem mass spectrometry (LC-MS/MS) analysis. KAT8-mediated acetylation of ATP5B at K201 impaired mitochondrial respiration and energy metabolism in AC16 cells. These contradictory findings, supported by both loss- and gain-of-function analyses, emphasize the importance of balanced regulation of mitochondrial protein acetylation by KAT8 in maintaining cardiac function.

## AGING AND ACETYLTRANSFERASES

Although cellular senescence, a state of persistent cell cycle arrest, may primarily serve as a cardioprotective mechanism to preserve functional cardiomyocytes, senescent cells also release pro-inflammatory cytokines, collectively known as the senescence-associated secretory phenotype (SASP). This SASP contributes to inflammation, fibrosis, and various cardiovascular diseases, including cardiac hypertrophy, diastolic dysfunction, and atherosclerosis^[245]^. Aging in the myocardium is linked to a metabolic switch from oxidative phosphorylation to increased glycolysis as an energy source, which can drive cardiac hypertrophy^[188]^. While KATs are crucial regulators of the cell cycle and senescence in stem cells, their specific roles as senescence regulators in the heart remain largely underexplored.

Circulating levels of senescence-associated secretory phenotype (SASP) proteins, particularly their combined presence rather than a single SASP protein, strongly predict age-related adverse health outcomes^[246]^. Key SASP proteins include growth differentiation factor 15 (GDF15), TNF receptor superfamily member 6 (FAS), osteopontin (OPN), TNF receptor 1 (TNFR1), ACTIVIN A, chemokine ligand 3 (CCL3), and IL-15^[246]^. Furthermore, metabolism and cellular senescence are closely interconnected, though the underlying molecular mechanisms are not fully understood. Recent studies have highlighted the links between metabolism and senescence. Plasma metabolomic analysis in biological aging showed decreased β-hydroxybutyrate, possibly due to impaired β-oxidation, alongside increased levels of unsaturated fatty acids and acylcarnitines, which may contribute to oxidative damage and inflammation, as well as elevated gut-derived aromatic amino acid metabolites^[247]^. Another study using metabolomic analysis and circulating SASP/inflammation markers identified three metabolites: β-cryptoxanthin, prolylhydroxyproline, and eicosenoylcarnitine, as putative drivers of biological aging rather than chronological age, highlighting the crucial roles of metabolic pathways in human biological aging^[248]^.

Mice deficient in the DNA excision-repair gene Ercc1 show accelerated aging phenotypes. Dietary restriction mitigated DNA damage in this mouse model, preventing the decline in transcription and thereby enhancing healthspan and lifespan^[249]^. Similarly, supplementation with a nicotinamide adenine dinucleotide (NAD^+^) precursor or β-hydroxybutyrate reversed metabolic, mitochondrial, and transcriptional changes, likely through the activation of sirtuin 1, and improved overall health in *Csb*−/− mice^[250]^. Beyond deacetylation, recent studies highlight the important roles of acetyltransferases in aging phenotypes. In *Caenorhabditis elegans*, nuclear DNA damage promotes mitochondrial β-oxidation and increases lipolysis, reducing fat depots and leading to histone hyperacetylation through the activation of *MYS-1* (Tip60) via increased acetyl-CoA levels^[251]^. Polyunsaturated fatty acids, particularly arachidonic acid, are increased via Tip60 in response to DNA damage. These findings suggest that Tip60 plays a critical role in the metabolic-epigenetic axis, promoting age-associated decline.

### The MYST family-related senescence

The enzymatic activity of KAT6A is essential for preserving the proliferative capacity of hematopoietic and neural stem cells by directly binding to the promoter of p16^INK4a^ and silencing its expression^[20,252,253]^. A KAT activity-deficient mutant of KAT6A (KAT6A-G657E) increased the expression of p16^INK4a^, leading to early entrance into replicative senescence in blood precursors; this effect was reversed by genetic deletion of p16^INK4a^ in hematopoietic and neural progenitor cells in mice. In cancer, inhibition of KAT6A and KAT6B using small molecule compounds WM-8014 and WM-1119 induces cell cycle exit and cellular senescence in an INK4A/ARF-dependent manner^[254]^. Supporting these findings, a recent ongoing open-label, multicenter, phase 1 clinical trial demonstrated that a selective catalytic inhibitor of KAT6A and KAT6B, PF-07248144, shows a tolerable safety profile and durable antitumor effects in heavily pretreated ER^+^HER2^−^ metastatic breast cancer patients^[255]^. These findings indicate that targeting KAT6A and KAT6B may provide therapeutic benefits in cancer treatment. Beyond cancer, a genome-wide CRISPR-based screen identified KAT7 as a driver of cellular senescence in two types of human mesenchymal precursor cells (hMPCs) with pathogenic mutations found in accelerated aging diseases such as Werner syndrome and Hutchinson-Gilford progeria syndrome^[256]^. Inactivation of KAT7 decreased histone H3K14ac and decreased p15^INK4b^ expression, thereby delaying senescence in hMPCs.

### The GNAT family-related senescence

HAT1 expression declines with age, and heterozygous HAT1 KO mice exhibit a significantly shorter lifespan, accompanied by elevated levels of senescence markers, endogenous DNA damage, and reactive oxygen species and mitochondrial dysfunction across various organs^[257]^. Selective RNAi-mediated inhibition of KAT2A in human senescent cell lines reduced the levels of aging markers and increased cell proliferation and lifespan. This effect was associated with reduced histone H3K9ac and H3K18ac, rather than H4 acetylation, suggesting a specific role of KAT2A in modulating age-associated histone acetylation^[258]^.

### The P300/CBP family-related senescence

High-throughput screening using short hairpin (sh)RNA targeting all known epigenetic regulator proteins identified p300 as a key driver of replicative senescence in primary human cells^[259]^. Unlike its paralog CBP, p300 induces histone hyperacetylation, including H3K12ac, H3K18ac, and H3K23ac on H3.1, as well as H3K27ac and K36ac on H3.3, which drives a senescence-specific gene expression program. Depletion of p300 suppresses senescence-related gene expression. The epigenetic reader bromodomain-containing protein 4 (BRD4), a member of the bromodomain and extra-terminal (BET) family, binds to H3K27ac near the SASP genes, thereby promoting cellular senescence. Notably, high-throughput screening identified a small molecule that degrades BRD4, showing a senolytic effect^[260]^.

In line with these findings, a recent study using chromatin immunoprecipitation sequencing and RNA sequencing analyses of cardiomyocytes isolated from mice aged 2, 6, and 18 months showed that the transition from adulthood to old age is characterized by increased deposition of H3K27ac at the enhancer regions of genes involved in cardiac hypertrophy signaling and glycolysis^[261]^. This study revealed that aging-associated pseudohypoxia activates glycolysis through p300/CBP-mediated enhancement of glycolysis-related genes, including the *hexokinase-2* (*Hk2*) gene. Pharmacological inhibition of p300/CBP with C646 improved cardiac function in mice at the onset of aging. These findings indicate that p300/CBP mediates senescence and aging-related metabolic remodeling in the heart, particularly the expression of glycolysis-related genes through enhancer activation.

In contrast to these findings, another study demonstrated that CBP and p300 are downregulated in aging mouse aortas^[262]^. This downregulation of p300 is linked to reduced expression and acetylation of 8-Oxoguanine DNA Glycosylase (OGG1), leading to increased oxidative DNA damage, elevated p53 expression, deposition of extracellular matrix proteins, and vascular stiffness^[262]^. These findings indicate that CBP and p300 play protective roles against vascular aging. In line with these results, an RNA interference screen identified *CBP-1*, the ortholog of human acetyltransferase *CBP/p300*, as an essential regulator of the mitochondrial unfolded protein response (UPR^mt^) in *C. elegans*^[263]^. ChIP-sequencing revealed that CBP-1 mediates H3K18ac and H3K27ac at the loci of UPR^mt^ transcription factors, including *activating transcription factor associated with stress-1* (*ATFS-1*), thereby inducing UPR^mt^ in response to mitochondrial stress. Analysis of the human Genotype-Tissue Expression (GTEx) database shows that mRNA levels of *CBP/p300* positively correlate with UPR^mt^-related transcripts across various tissues and are associated with increased longevity. These findings indicate that CBP and p300 contribute to health and longevity by promoting the mitochondrial unfolded protein response and preventing vascular aging.

### NAT-related senescence

N-terminal acetylation was initially thought to promote ubiquitin-mediated proteolytic degradation^[264]^; however, recent studies have shown that N-terminal acetylation generally protects against protein degradation in human cells^[265]^ and *Saccharomyces cerevisiae*^[266]^. Consistent with these findings, a recent genome-wide CRISPR knockout screen revealed that the ubiquitin ligase complexes UBR4-KCMF1, UBR1, and UBR2 of the Arg/N-degron pathway recognize proteins that lack NatC-mediated N-terminal acetylation, promoting their degradation in human cells^[267]^. In *Drosophila*, NatC KO is associated with male sterility, reduced longevity, and age-dependent loss of motility, effects that were reversed by inhibition of UBR. These findings suggest that NatC-mediated N-terminal acetylation enhances protein stability by shielding proteins from proteasomal degradation, serving as a protective mechanism against aging-related protein degradation.

## CONCLUSIONS AND PERSPECTIVES

The development and advancement of omics techniques have uncovered previously unrecognized acetylation sites in histone and non-histone proteins, providing new insights into their regulatory mechanisms. Concurrently, significant progress has been made in elucidating the molecular mechanisms by which acetyltransferases regulate cellular processes such as metabolism, proliferation, apoptosis, and senescence, all of which are central to the pathogenesis of cardiovascular disease and aging at the organ level. However, several key questions remain unanswered. First, while clinical studies provide strong evidence on the impact of healthy and unhealthy diets on cardiovascular health and associated epigenomic modifications, it remains unclear whether acetyltransferases directly contribute to the beneficial effects of healthy diets or exercise. If so, identifying which specific acetyltransferases are involved and understanding how they mediate these effects are essential areas for future research. Second, although mass spectrometry analyses have identified PTMs on multiple acetyltransferase residues, the impact of these modifications on acetyltransferase function is still largely unknown. The generation of site-specific acetylation-resistant and acetylation-mimic mutants would provide a clear strategy to explore these questions both *in vitro* and *in vivo* models.

Recent studies have emphasized the vital roles of acetyltransferases in mitochondrial function, DNA damage repair, inflammation, and autophagy. However, their involvement in the pathogenesis of heart failure with preserved ejection fraction (HFpEF), a condition characterized by chronic inflammation and altered metabolism, remains poorly understood. Given that chronic inflammation is a systemic condition, inhibiting specific acetyltransferases solely in the heart may not provide effective cardioprotection. In this regard, it is essential to consider the effects of acetyltransferases on other tissues and cell types, including immune cells, in the context of HFpEF. Additionally, while KATs are known to be crucial for HSC maintenance, their potential roles in cardiac senescence or senolysis, and their regulatory functions in cardiomyocyte aging, remain largely uncharacterized.

Moreover, various KATs exhibit significant changes in expression and, presumably, function in cardiometabolic disease, including ischemic and hypertensive heart disease and diabetes. However, the functional roles and significance of these changes remain largely unexplored. It is also important to note that deacetylates may counteract the effects of acetyltransferase, making it crucial to distinguish the impacts of specific acetylation events from the overall function of acetyltransferases. Furthermore, acetyltransferases may serve non-catalytic roles, such as acting as scaffold proteins in complex formation, independent of their enzymatic activity. Catalytic-dead mutants could help delineate the enzymatic functions from those observed in KO models.

Based on the previous findings and experiments, we propose the following experimental approaches to explore the mechanisms in greater depth: dietary intervention, intermittent fasting, exercise, and modulation of metabolites and cofactors in combination with genetic manipulation in mice.

To investigate the influence of specific dietary macronutrients or components on acetyltransferase activity, dietary interventions combined with omics analyses will be valuable: (1) A high-fat diet compared to a normal-fat diet, where a high-fat diet increases acetyl-CoA levels in the heart; and (2) A low-carbohydrate high-fat ketogenic diet compared to a high-carbohydrate diet, where physiological ketosis increases NAD^+^ levels. Notably, ketone body supplementation can increase acetylation levels in the heart.

To investigate the effects of lifestyle modifications beyond dietary interventions, intermittent fasting and exercise can be applied, which increase NAD^+^ levels while reducing acetyl-CoA levels, thereby modulating acetyltransferase activity. The advantage of these strategies lies in their physiological relevance. However, it is important to note that the effects of these interventions may not be solely attributable to changes in NAD^+^ and acetyl-CoA levels, as other systemic factors could also be involved.

To investigate the direct impact of NAD^+^ and acetyl-CoA levels on cardiometabolic health and aging, interventions such as NAD^+^ supplementation, its precursors, acetyl-CoA supplementation, NAD^+^ depletion, and acetyl-CoA depletion may serve as appropriate approaches. These methods will influence the overall activities of acetyltransferases or deacetylases. Additionally, pharmacological inhibition of lysine deacetylases, such as Valproic acid, butyric acid, nicotinamide, or SCFAs, could provide further insight into the role of deacetylases in these processes. For more targeted investigations, genetic overexpression or inhibition of specific acetyltransferases can be employed. If the target acetylation site is identified, site-specific inhibition using knock-in approaches will allow for the determination of the precise role of acetyltransferase-mediated acetylation *in vivo*. These approaches, when combined with dietary, metabolic, and physical activity interventions, should provide a more comprehensive understanding of the specific effects of acetyltransferases on cardiometabolic health and aging.

In summary, future research is needed to unravel the complex roles of acetyltransferases in cardiovascular disease and aging, with the goal of developing novel pharmacopreventive strategies that specifically target the signaling pathways related to acetyltransferases, identifying biomarkers for the prediction of adverse cardiovascular events, and tailoring personalized lifestyle interventions based on these biomarkers.

## Figures and Tables

**Table 1.** Acetyltransferases categorized by their families and respective members. The table includes the known histone and non-histone targets discussed in the main text, along with their subcellular localization, associated mouse models, and observed phenotypes

Family	Enzyme	Histone targets	Non-histone targets	Subcellular localization	Mouse model	Phenotype	Ref.

MYST (canonical)	KAT6A (MOZ) (MYST3)	H3K9, H3K14, H3K23	p53	Nucleus	MOZ^−/−^	Embryonic lethality (E15), defect of HSCs	[19]
					MOZ-G657E kinase dead	Marked reductions in HSC number	[20]
					C-terminal truncated mutant	Die at birth due to aortic arch defects. Severe dysgenesis of both the thymus and spleen. HSC defects. Like KAT6A syndrome	[21]
						H3K9 hypoacetylation, Hox gene repression	[22]
					Lck-Cre	Reduced H3K9ac at *CD8* gene locus and reduced *Cd8a* gene	[23]
					NEX(^F/+^)-Cre	Impaired synaptic structure and plasticity in hippocampal CA3, memory deficits	[24]
	KAT6B (MORF) (MYST4)	H3K9, H3K14, H3K23		Nucleus	Qkf ^gt/+^	MAPK activation, Noonan syndrome-like phenotype	[27]
					Qkf ^gt/+^	Craniofacial defects, developmental defects in central nervous	[28]
					KAT6B^−/−,+/−^	Die before weaning (^−/−^), like SBBYSS and GPS (^+/−^ )	[31]
	Tip60 (KAT5)	H3K14, H4K5/8/12/16	p53, c-Myc, Rheb, ULK1, Pacer	Nucleus	Tip60^−/−^	Embryonic lethality near the blastocyst stage	[38]
					Tip60-KD	Impaired DNA repair and apoptosis response	[41]
					Nes-Cre	Perinatal lethality. Brain hypoplasia and microcephaly	[44]
					Myh6-MCM	Better cardiac function post-MI	[232]
	KAT8 (MOF) (MYST1)	H4K16	p53, Nrf2, TIP5, IRF3, ATP5B	Nucleus mitochondria	KAT8^−/−^	Failure to develop beyond the expanded blastocyst stage	[53,54]
					Vav1-Cre	Lethal hematopoietic failure at an early postnatal stage	[55]
					Mx1-Cre	Dramatic hematopoietic failure	[55]
					Emx1-Cre	Cerebral hypoplasia, apoptosis, early lethality before weaning	[57]
					Krt14-Cre	Lethality within hours after birth due to severe defects of the skin	[242]
	HBO1 (KAT7) (MYST2)	H3K14, H4K5/8/12	ORC2, MCM2, CDC6	Nucleus cytoplasm	HBO1^−/−^	Cell death and DNA fragmentation. Embryonic lethality	[59]
					Mx1-Cre	Hematopoietic failure with pancytopenia	[65]
					Mx1-Cre	Survival advantage in leukaemic cells	[65]
GNAT (canonical)	HAT1 (KAT1)	H2AK5, H4K5, H4K12	CBP, HBO1, AGK, DECR1 ATP5B	Nucleus Cytoplasm mitochondria	HAT1^−/−^	Neonatal lethality due to severe defects in lung development. Craniofacial defects with abnormalities in skull and jaw	[72]
					HAT1^+/−^	Early-onset aging. Kyphosis, muscle atrophy, ROS	[257]
	KAT2A (GCN5)	H3K9, H3K14,	c-Myc, PGC-1α, C/EBPα, α-tubulin	Nucleus cytoplasm	KAT2A^−/−^	Embryonic lethality (days 9.5~11.5), failure to form dorsal mesoderm lineages due to severe apoptosis	[79]
					CamKIIa-Cre	Impaired hippocampus-dependent memory consolidation	[42]
					Mx1-Cre	Depletion of AML stem cells, imposing a delay to disease initiation and severely impairing AML propagation, specific loss of H3K9Ac	[83]
	KAT2B (PCAF)	H3K9, H3K14	p53, c-Myc, FoxP3	Nucleus cytoplasm	KAT2B^−/−^	Viable with no obvious detrimental phenotype	[80]
					KAT2B^−/−^	Short-term memory deficits, hippocampal alterations, fear to stress	[88,89]
					KAT2A-Villin-Cre & KAT2B^−/−^	Mitochondrial double-stranded RNA accumulation, impaired intestinal stem cell renewal, lethality in a week after deletion	[81]
p300/CBP (canonical)	CBP (KAT3A)	H3K18, H3K27	p53, NF-κB, Stat1, Ku70, YAP	Nucleus cytoplasm	CBP^−/−^	Embryonic lethality (E10.5-E12.5) due to massive hemorrhage, developmental delays, impaired hematopoiesis	[100]
					CBP^+/−^	Skeletal abnormality. Like Rubinstein-Taybi syndrome	[95]
	p300 (KAT3B)	H2A, H2B, H3K12, H3K18, H3K23, H3K27	NF-κB, c-Myc, raptor, androgen receptor, Beclin1, p53	Nucleus cytoplasm	p300^−/−^	Embryonic lethality (E9–11.5), defects in neurulation, cell proliferation, and heart development	[96]
Noncanonical KATs	TAF1 (KAT4)	(TFIID complex formation)		Nucleus	TAF1^+/−^(F) TAF1^−/Y^ (M)	KO male: Embryonic lethality. TAF1^+/−^ female: Increase in weight, reduced movement	[115]
	ELP3 (KAT9)	H3, H4 (transcription elongation)	α-Tubulin	Nucleus Cytoplasm	ELP3^−/−^	ELP3 ^−/−^ : Embryonic lethality (E8). Mediate amyotrophic lateral sclerosis	[120]
	GTF3C4 (KAT12)	H3 (TFIIIC2 complex)		Nucleus			
